# Supplementary material for: Dynamic Heterogeneity and DNA Methylation in Embryonic Stem Cells
Source: Mol Cell. 2014 Jul 17;55(2):319–31. doi: 10.1016/j.molcel.2014.06.029 (PMC4104113; doi:10.1016/j.molcel.2014.06.029)
Supplement: Document S1. Supplemental Experimental Procedures, Figures S1–S5, and Table S1 [file mmc1.pdf]

Molecular Cell, Volume *55*

Supplemental Information

Dynamic Heterogeneity and DNA Methylation in Embryonic Stem Cells

Zakary S. Singer, John Yong, Julia Tischler, Jamie A. Hackett, Alphan Altinok, M. Azim Surani, Long Cai, and Michael B. Elowitz

Figure S1

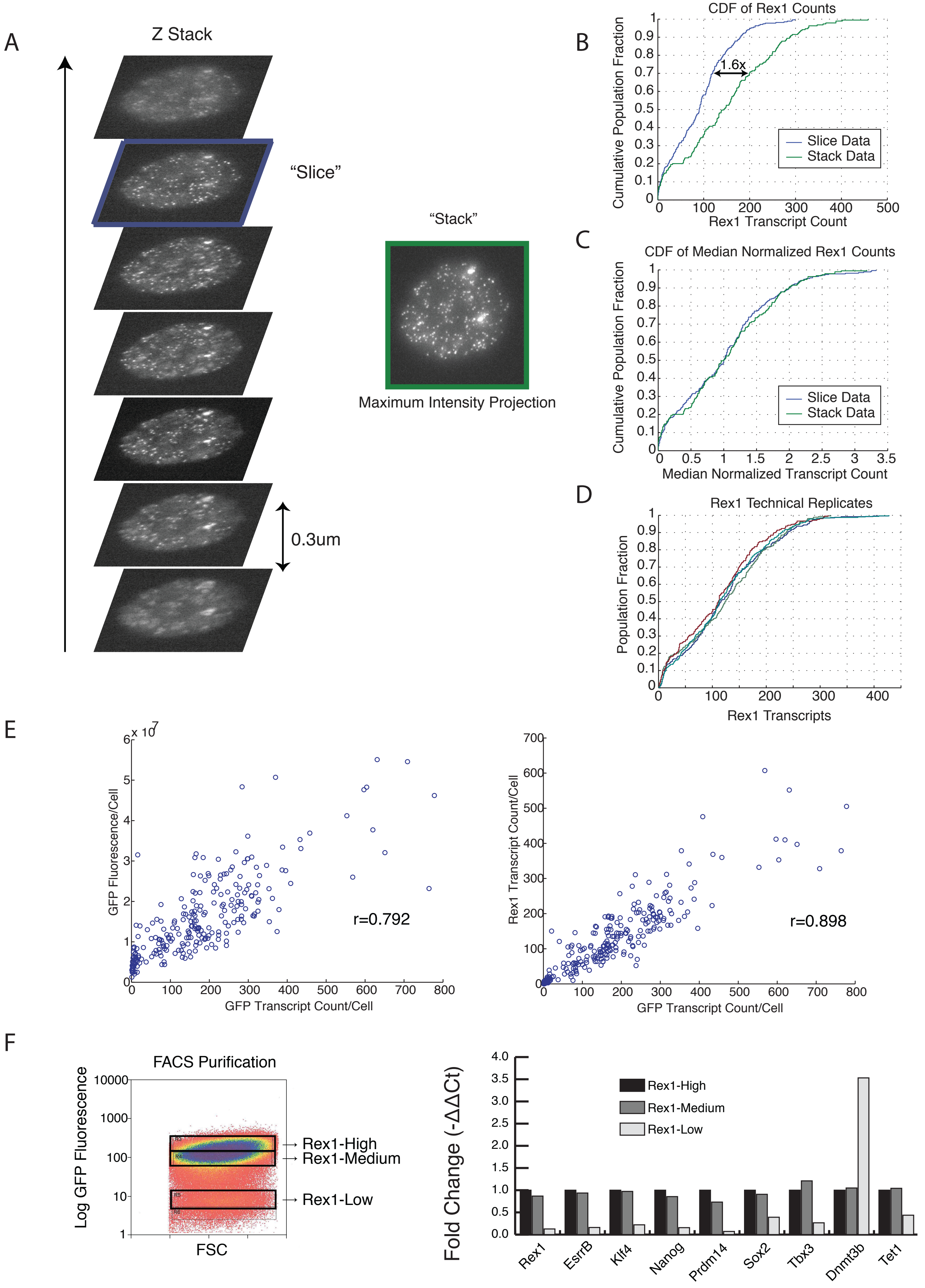

A
Z Stack
"Slice"
"Stack"
Maximum Intensity Projection
0.3um
B
CDF of Rex1 Counts
Cumulative Population Fraction
Rex1 Transcript Count
1.6x
Slice Data
Stack Data
C
CDF of Median Normalized Rex1 Counts
Cumulative Population Fraction
Median Normalized Transcript Count
Slice Data
Stack Data
D
Rex1 Technical Replicates
Population Fraction
Rex1 Transcripts
E
x 10^7
GFP Fluorescence/Cell
GFP Transcript Count/Cell
r=0.792
Rex1 Transcript Count/Cell
GFP Transcript Count/Cell
r=0.898
F
FACS Purification
Log GFP Fluorescence
FSC
Rex1-High
Rex1-Medium
Rex1-Low
Fold Change (-ΔΔCt)
Rex1-High
Rex1-Medium
Rex1-Low
Rex1
EsrrB
Klf4
Nanog
Prdm14
Sox2
Tbx3
Dnmt3b
Tet1

Figure S2.

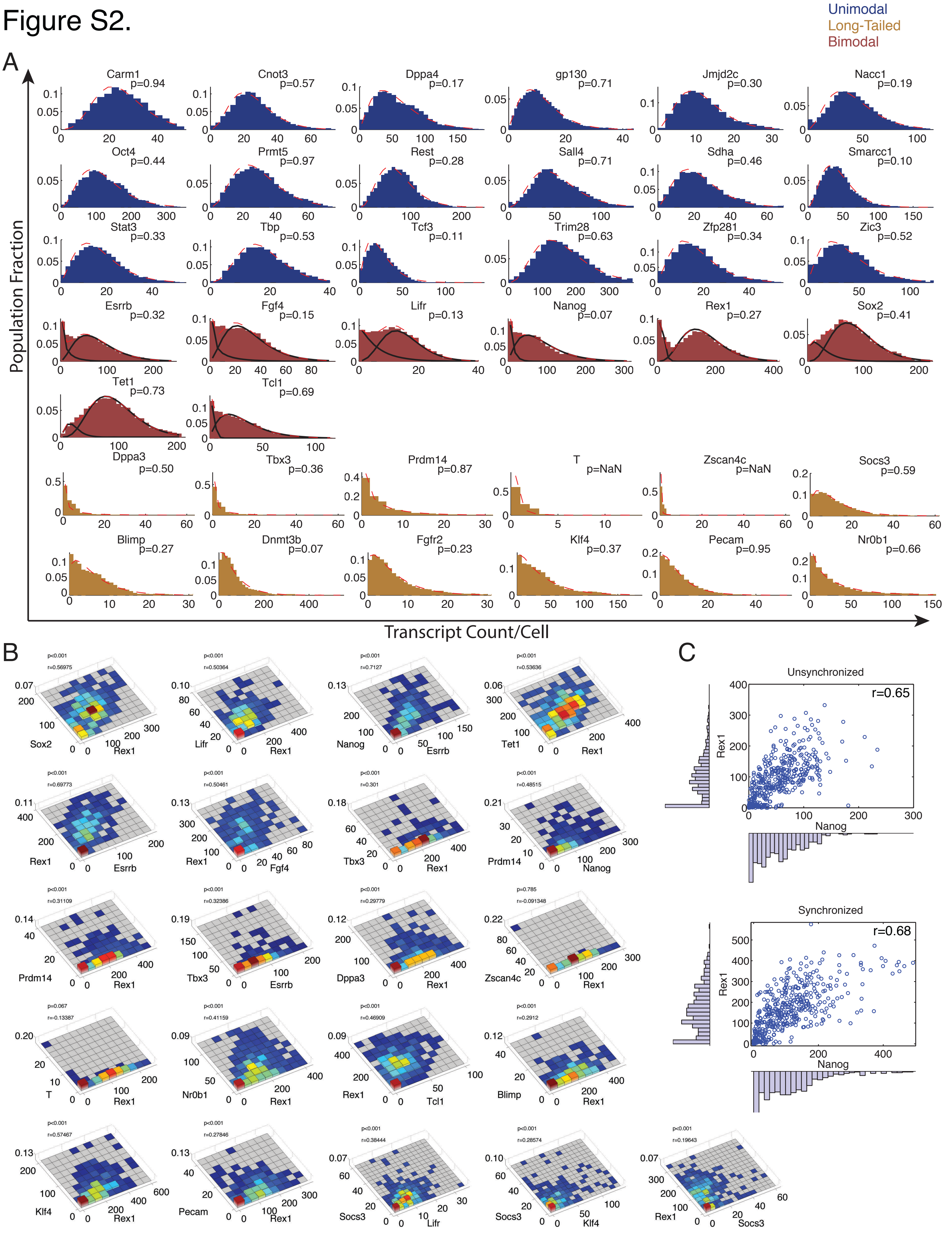
Unimodal
Long-Tailed
Bimodal
A
Population Fraction
Transcript Count/Cell
Carm1 p=0.94
Cnot3 p=0.57
Dppa4 p=0.17
gp130 p=0.71
Jmjd2c p=0.30
Nacc1 p=0.19
Oct4 p=0.44
Prmt5 p=0.97
Rest p=0.28
Sall4 p=0.71
Sdha p=0.46
Smarcc1 p=0.10
Stat3 p=0.33
Tbp p=0.53
Tcf3 p=0.11
Trim28 p=0.63
Zfp281 p=0.34
Zic3 p=0.52
Esrrb p=0.32
Fgf4 p=0.15
Lifr p=0.13
Nanog p=0.07
Rex1 p=0.27
Sox2 p=0.41
Tet1 p=0.73
Tcl1 p=0.69
Dppa3 p=0.50
Tbx3 p=0.36
Prdm14 p=0.87
T p=NaN
Zscan4c p=NaN
Socs3 p=0.59
Blimp p=0.27
Dnmt3b p=0.07
Fgfr2 p=0.23
Klf4 p=0.37
Pecam p=0.95
Nr0b1 p=0.66
B
C
Unsynchronized
r=0.65
Rex1
Nanog
Synchronized
r=0.68
Rex1
Nanog

Figure S3

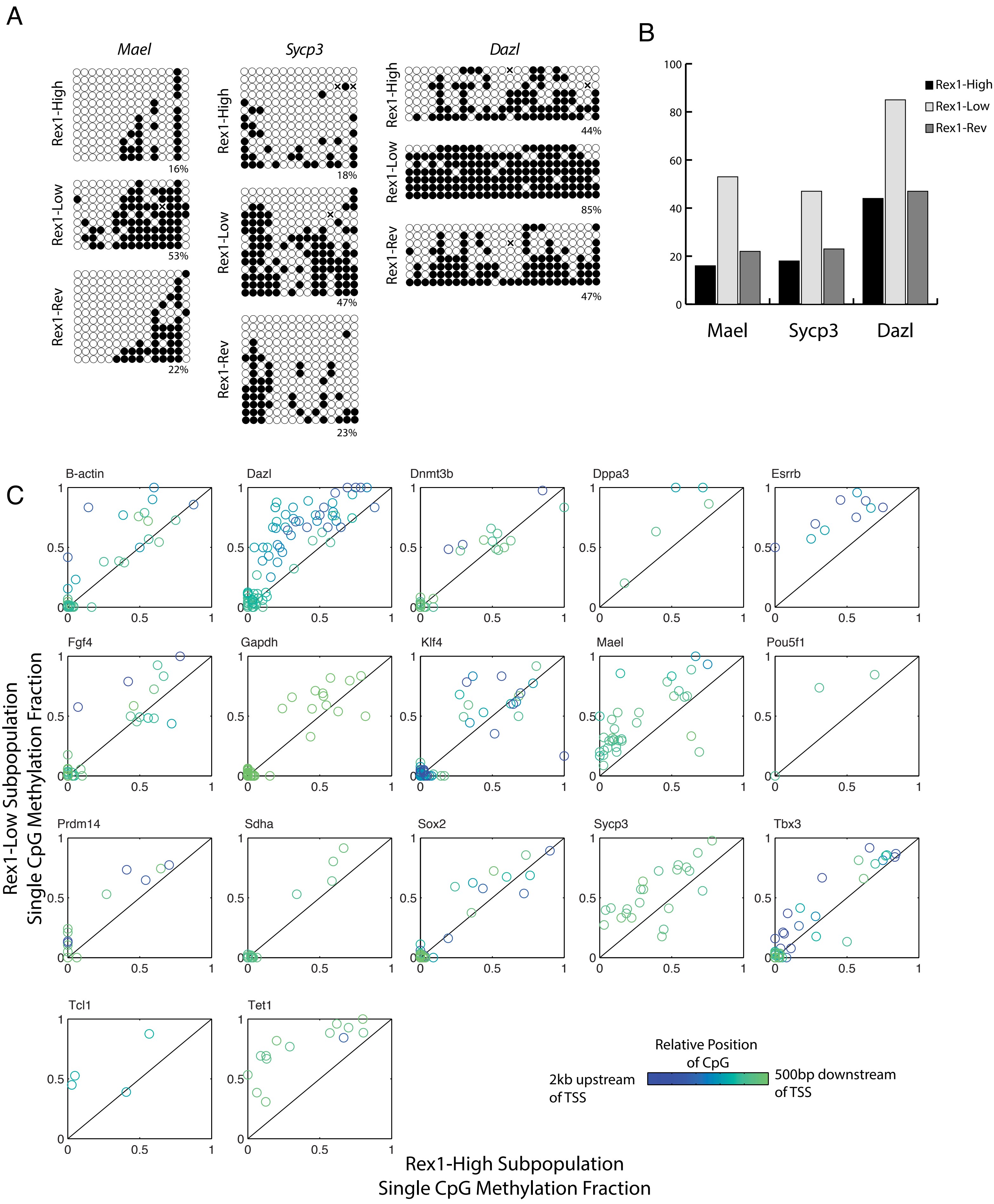

A
Mael
Sycp3
Dazl
Rex1-High
Rex1-Low
Rex1-Rev
16%
53%
22%
18%
47%
23%
44%
85%
47%
B
Rex1-High
Rex1-Low
Rex1-Rev
Mael
Sycp3
Dazl
C
B-actin
Dazl
Dnmt3b
Dppa3
Esrrb
Fgf4
Gapdh
Klf4
Mael
Pou5f1
Prdm14
Sdha
Sox2
Sycp3
Tbx3
Tcl1
Tet1
Rex1-Low Subpopulation
Single CpG Methylation Fraction
Rex1-High Subpopulation
Single CpG Methylation Fraction
Relative Position
of CpG
2kb upstream
of TSS
500bp downstream
of TSS

Figure S4.

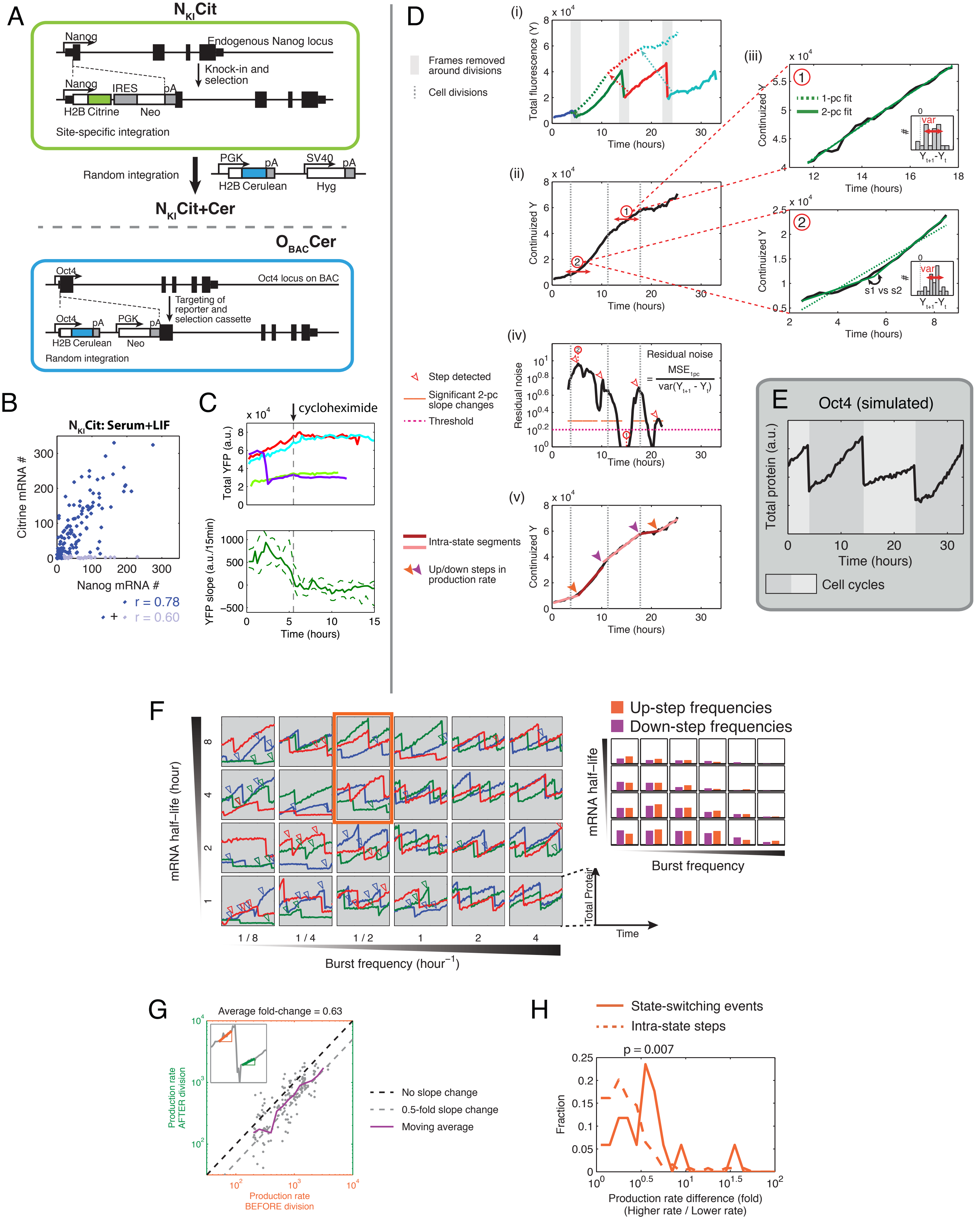

A
N_KI Cit
Nanog
Endogenous Nanog locus
Knock-in and selection
Nanog
IRES
pA
H2B Citrine
Neo
Site-specific integration
Random integration
PGK
pA
SV40
pA
H2B Cerulean
Hyg
N_KI Cit+Cer
O_BAC Cer
Oct4
Oct4 locus on BAC
Targeting of reporter and selection cassette
Oct4
pA
PGK
pA
H2B Cerulean
Neo
Random integration
B
N_KI Cit: Serum+LIF
Citrine mRNA #
Nanog mRNA #
r = 0.78
r = 0.60
C
cycloheximide
Total YFP (a.u.)
YFP slope (a.u./15min)
Time (hours)
D
Frames removed around divisions
Cell divisions
(i)
Total fluorescence (Y)
Time (hours)
(ii)
Continuized Y
Time (hours)
(iii)
1-pc fit
2-pc fit
var
Continuized Y
Time (hours)
s1 vs s2
(iv)
Step detected
Significant 2-pc slope changes
Threshold
Residual noise
Residual noise
MSE_1pc
var(Y_t+1 - Y_t)
Time (hours)
(v)
Intra-state segments
Up/down steps in production rate
Continuized Y
Time (hours)
E
Oct4 (simulated)
Total protein (a.u.)
Time (hours)
Cell cycles
F
mRNA half-life (hour)
Burst frequency (hour^-1)
1 / 8
1 / 4
1 / 2
1
2
4
Total Protein
Time
Up-step frequencies
Down-step frequencies
mRNA half-life
Burst frequency
G
Average fold-change = 0.63
Production rate AFTER division
Production rate BEFORE division
No slope change
0.5-fold slope change
Moving average
H
State-switching events
Intra-state steps
p = 0.007
Fraction
Production rate difference (fold)
(Higher rate / Lower rate)

Figure S5.

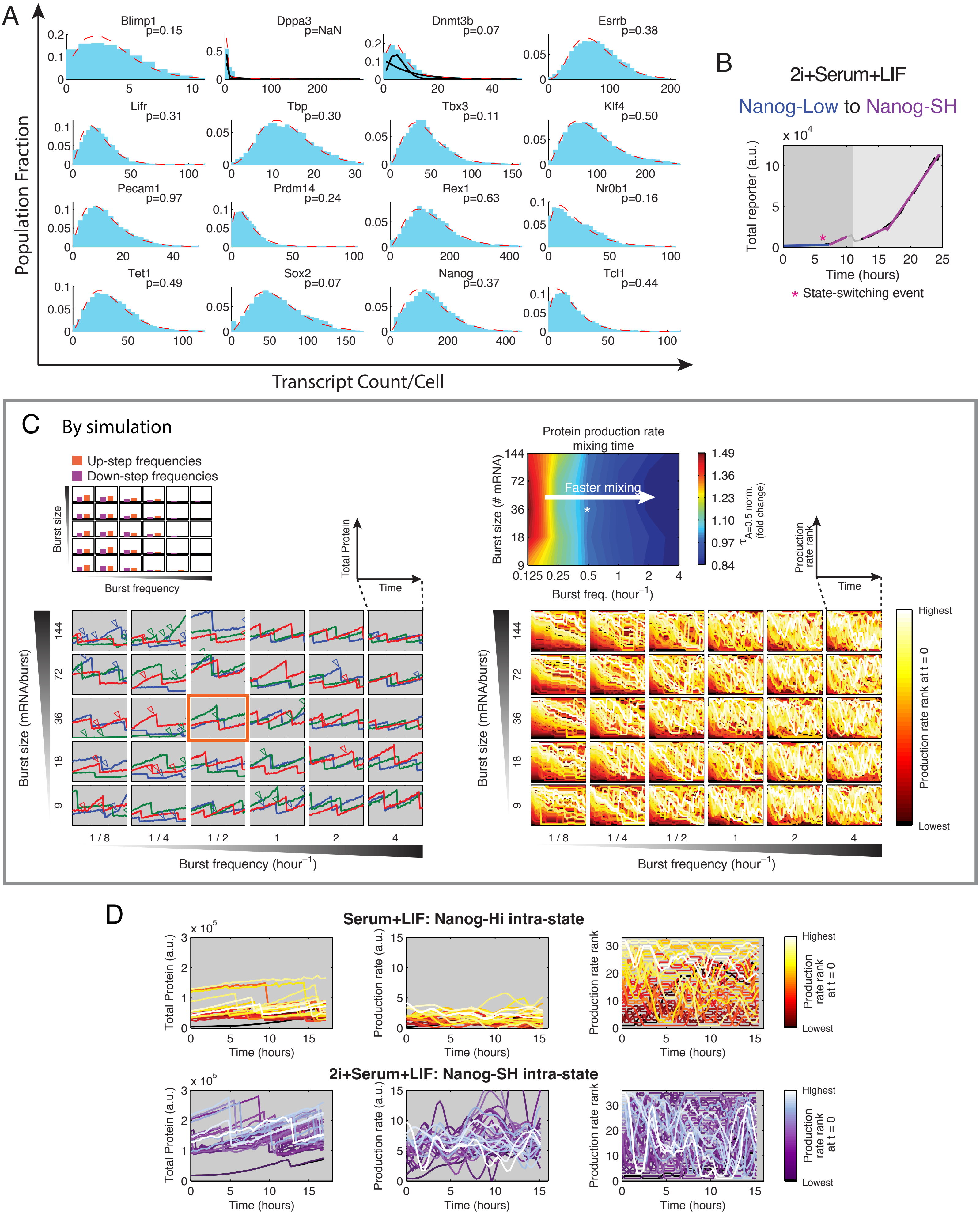
A
Population Fraction
Transcript Count/Cell
Blimp1 p=0.15
Dppa3 p=NaN
Dnmt3b p=0.07
Esrrb p=0.38
Lifr p=0.31
Tbp p=0.30
Tbx3 p=0.11
Klf4 p=0.50
Pecam1 p=0.97
Prdm14 p=0.24
Rex1 p=0.63
Nr0b1 p=0.16
Tet1 p=0.49
Sox2 p=0.07
Nanog p=0.37
Tcl1 p=0.44
B
2i+Serum+LIF
Nanog-Low to Nanog-SH
Total reporter (a.u.)
Time (hours)
* State-switching event
C
By simulation
Up-step frequencies
Down-step frequencies
Burst size
Burst frequency
Total Protein
Time
Burst size (mRNA/burst)
Burst frequency (hour^{-1})
Protein production rate mixing time
Faster mixing
Burst size (# mRNA)
Burst freq. (hour^{-1})
$\tau_{A=0.5}$ norm. (fold change)
Production rate rank
Production rate rank at t = 0
Highest
Lowest
D
Serum+LIF: Nanog-Hi intra-state
2i+Serum+LIF: Nanog-SH intra-state
Total Protein (a.u.)
Production rate (a.u.)
Production rate rank
Time (hours)
Production rate rank at t = 0

Table S1. State-switching events show no correlation between sister cells

# of sister pairs* / Direction of switch	Neither sister switched	Only one sister switched	Both sisters switched	Expected number of sister pairs that both switched [95% C.I.]**
N^{Lo} to N^{Hi}	169	7	0	**[0 – 1]**
N^{Hi} to N^{Lo}	139	15	2	**[0 – 2]**

Data shown are combined results from two independent experiments. Analysis of individual data sets yields the same conclusion.

* Data points are discarded if one of the cells in a sister pair was lost or not traceable in the movie

** Confidence interval obtained by random permutation test with 100,000 trials. Green indicates observed frequency of sister pairs in which both cells switched falls within the 95% C.I.

Supplemental Figure, Table and Movie Legends

Figure S1. Validation of smFISH; related to Figure 1

(A) A stack of snapshots taken through the whole volume of a single cell; the resulting maximum-intensity projection (green box), and a single slice (blue box) are fed into the image-processing algorithm for dot-detection.

(B) Cumulative Distributions of dot counts for each of the two imaging approaches is shown across a population of cells.

(C) Same distributions as in B, but normalized by the sample median.

(D) Technical replicates for the single-slice approach.

(E) Correlation between dGFP protein fluorescence as measured simultaneously with *dGFP* transcripts. (left), and correlation between *Rex1* (unmodified allele) and *dGFP* (knock-in reporter on second allele) transcripts (right). r is the Pearson correlation coefficient.

(F) (Left) Sorted subpopulations of the bimodal Rex1-*d*GFP knock-in reporter. (Right) qPCR results on these subpopulations for a subset of target genes also examined by smFISH. Values were normalized to expression levels of the housekeeping gene *Gapdh*, and are represented as 2^(-ΔΔCt) with respect to the '*Rex1*-high' subpopulation

Figure S2. mRNA distributions and correlations by smFISH; related to Figure 2

(A) Empirical distributions and MLE fits for unimodal, bimodal, and long-tailed genes. *p*-values are for χ^2 GOF tests. $p>0.05$ indicates that the fit to the distribution is indistinguishable from the empirically measured distribution. Where present, solid lines represent components of the fit. Dashed line represents the overall fit to the distribution.

(B) Pairwise relationships between heterogeneously expressed genes. *p*-values are from the 2D KS-test. *r* is the Pearson correlation coefficient.

(C) Correlation and marginal distributions of *Rex1* and *Nanog* in a control population (top) and population synchronized by a double thymidine block fixed immediately following the block (bottom). r

is the Pearson correlation coefficient.

Figure S3. Differential methylation between *Rex1* states; related to Figure 3

(A) Locus specific bisulfite sequencing plots between *Rex1*-high, -low, and -low-to-high-reverting cells at three targets of methylation. Open circles are unmethylated, filled circles are methylated, and x's are unknown.

(B) Measurements from A are plotted as bar graphs for comparison.

(C) Scatter plots showing how single CpGs in the promoters of a given gene change between *Rex1*-high and -low states. Color coding represents the position of a base relative the transcriptional state site.

Figure S4. Construction and analysis of live cell reporters, and simulations based on observed kinetics; related to Figure 4

(A) Schematic of *Nanog* reporter (top) and *Oct4* reporter (bottom) construction.

(B) Correlation between *Nanog* (unmodified allele) and *Citrine* (knock-in reporter on second allele) transcripts in N_{KI}Cit cell line. r, Pearson correlation coefficient. Light blue, presumed fraction of cells with silenced reporter cassettes (~10% of all cells; see Supp. Info. for discussion); dark blue, remaining cell population.

(C) H2B-Citrine protein degradation rate assayed by blocking translation during movie at time indicated. Total YFP became flat (top) with negligible slope (bottom) shortly after cycloheximide treatment.

(D) Identification of sharp inflections in total fluorescence traces. (i) First, frames around cell divisions are removed and fluorescence lost during divisions is added back to the daughter trace to create a continuous trace for each lineage (ii), where a step detector spanning a 6-hour window is applied across consecutive frames. (iii) For each window, a one-piece linear fit is compared with a two-piece fit that is flexible at the midpoint. A two-piece fit is considered better than a one-piece fit when two criteria are

met: 1) Residual noise of the one-piece fit is higher than a threshold, see Supp. Info.), and 2) the slopes of the two-piece fit are significantly different between the two pieces. iv) For each stretch of frames meeting both criteria 1 (magenta line indicates threshold) and 2 (orange line indicates where two-piece fit yields significantly different slopes), the window with the highest residual noise is assigned to be the inflection. v) Continuized trace approximated into linear segments between identified points of inflection.

(E) Apparent steps from simulated Oct4 expression under the bursty transcription model using parameters estimated from smFISH.

(F) Protein traces were simulated under the bursty transcription model over various mRNA half-life and burst frequency combinations; mean burst size was kept constant at 35 mRNA/burst. Gaussian noise proportional to the total protein level and equivalent to the magnitude of frame-to-frame variation empirically observed was added to the simulated traces for comparability. Arrowheads indicate detected steps on simulated trace of the corresponding color. Note that changes in production rate around cell division events can be identified as steps either before or after the division. Red box: Estimated regime for Nanog-Hi in serum+LIF. Right: Variation in the frequency of detected steps over the same parameter space.

(G) Production rates decrease by an average of 0.63-fold across cell divisions. Each point represents a division event. Average production rates of the 4-hour windows before and after each cell division are compared. Black dotted line: zero change; grey dotted line: 0.5-fold change; purple line: average trend; Inset: example trace indicating slope before and after division.

(H) Changes in production rate over state-switching events or intra-state steps. "Higher rate"-to-"lower rate" ratios are plotted for all steps and events, i.e. down-steps and *Nanog*-high-to-*Nanog*-low switching events are represented by the reciprocals of rate change. (*p*-value, KS test)

Figure S5. Quantitative analysis of how 2i+serum+LIF affect static distributions and dynamics of

gene expression for pluripotency regulators; related to Figure 5

(A) smFISH transcript count distribution of factors in 2i+serum+LIF with MLE fits overlaid. p-values are for χ^2 GOF tests. p>0.05 indicates that the fit to the distribution is indistinguishable from the empirically measured distribution. Where present, solid lines represent components of the fit. Dashed line represents the overall fit to the distribution.

(B) Example trace of cells switching from *Nanog*-low to *Nanog*-SH in 2i+serum+LIF.

(C) Left: simulated traces similar to Fig. S4F, except over various combinations of burst size and burst frequency; mRNA half-life was kept constant at 4 hours. Bottom right: rank of production rate of 30 randomly selected traces (out of a total of 200) in each simulation under the corresponding parameter combination. Traces are color-coded by the initial rank at t = 0 as in D. Top right: mixing time of protein production rate, defined as the time where auto-correlation of rank drops below 0.5.

(D) *Nanog* expression dynamics of cells in serum/LIF with or without 2i. Each trace represents one cell randomly picked from a tracked lineage tree. Production rates are normalized by cell size and ranked within the group for each time point. Traces are color-coded by the initial rank at t = 0.

Table S1. State-switching events show no correlation between sister cells; related to Figure 4

Movie S1. *Nanog*-high to *Nanog*-low switch in serum+LIF; related to Figure 4

Cells imaged in serum+LIF condition. Shown are examples of cells switching from *Nanog*-high to *Nanog*-low.

Movie S2. *Nanog*-low to *Nanog*-high switch in serum+LIF; related to Figure 4

Cells imaged in serum+LIF condition. One of the lineages switched from *Nanog*-low to *Nanog*-high.

Movie S3. *Nanog*-SH cells in 2i+serum+LIF; related to Figure 5

Cells imaged in 2i+serum+LIF condition. Nanog reporter expression is homogeneous compared to cells grown without 2i.

Movie S4. *Nanog*-low to *Nanog*-SH switch in 2i+serum+LIF; related to Figure 5

Cells imaged in 2i+serum+LIF condition. Shown are *Nanog*-low cells which were rare and switched to *Nanog*-SH.

Supplemental Experimental Procedures

Detailed Culture Conditions

All cells were maintained in humidity-controlled chamber at 37ºC, with 5% CO_2 in serum+LIF media [Glasgow Minimum Essential Medium (GMEM) supplemented with 10% FBS (HyClone, Thermo Scientific), 2 mM glutamine, 100 units/ml penicillin, 100 ug/ml streptomycin, 1 mM sodium pyruvate, 1000 units/ml Leukemia Inhibitory Factor (LIF, Millipore), 1X Minimum Essential Medium Non-Essential Amino Acids (MEM NEAA, Invitrogen) and 50 uM β-Mercaptoethanol.

Correlation between *Citrine* and *Nanog* transcripts in *Nanog* knock-in reporter cells (N_{KI}Cit)

We validated the *Nanog* knock-in reporter by performing smFISH for correlation between *Nanog* (unmodified allele) and *Citrine* (knock-in reporter on second allele) (Fig. S4B). We observed that when grown in serum+LIF conditions, ~10% of cells contained *Nanog* but no *Citrine* transcripts, likely due to silenced expression of their reporter cassettes during prolonged propagation without antibiotics. The remaining cell population showed even stronger correlation between *Nanog* and *Citrine* transcripts ($r = 0.78$). We corrected for the potential systematic error that may result in the calculation of low-to-high switching rate such that an observed rate of 1.9 ± 0.29 transitions per 100 cell cycles was adjusted to the reported 2.3 ± 0.25 (mean ± SD). We note that the magnitude of this error does not alter key conclusions, including those about the relative stabilities of the two states. Furthermore, the asymmetry of this silencing behavior – we did not find a corresponding fraction of cells expressing *Citrine* but no *Nanog* transcripts – suggests that this is unlikely a result of mono-allelic regulation.

smFISH Procedure and imaging system

Up to 48 20mer DNA probes per target mRNA were synthesized and conjugated to Alexa fluorophore 488, 555, 594, or 647 (Life Technologies) and then purified by HPLC. Cells for smFISH experiments were plated at 40,000/cm^2 and harvested after 48 hours. Trypsinized cells were washed in PBS and fixed in 4% formaldehyde at room temperature for 5 mins. Fixed cells were resuspended in 70% ethanol and

stored at -20°C overnight. The next day, cells were hybridized with 4nM probe per target species at 30°C, in 20% Formamide, 2X SSC, 0.1g/ml Dextran Sulfate, 1mg/ml E.coli tRNA, 2mM Vanadyl ribonucleoside complex, and 0.1% Tween 20 in nuclease free water. The following morning, cells were washed in 20% Formamide, 2x SSC, and 0.1% Tween 20 at 30°C, followed by two washes in 2x SSC + 0.1% Tween 20 at room temperature. Hybridized cells were placed between #1 coverslips and flattened by applying pressure evenly across the glass.

After flattening cells between coverslips, dots typically span two distinct focal planes. However, to maximize the number of cells imaged in a given acquisition time, only one of these focal planes was captured. This results in approximately ~60% of each cell's transcripts being captured in a single slice, as compared to taking a stack of images across the entire volume of each cell (Fig. S1A-D).

Imaging was performed on a Nikon Ti-E with Perfect Focus, Semrock FISH filtersets, Lumencor Sola illumination, 60x 1.4NA oil objective, and a Coolsnap HQ2 camera. Snapshots were taken using an automated grid-based acquisition system on a motorized ASI MS-2000 stage.

Monte-Carlo Bivariate Kolmogorov-Smirnov Test

The 1D Kolmogorov-Smirnov test was extended to 2D dimensions (Peacock, 1983) to determine whether an empirical bivariate distribution showed any dependence between variables; the 2D Cumulative Distribution Function (CDF) is computed in each possible quadrant of the 2D plane $P(x<x0)$, $P(y<y0)$; $P(x>x0)$, $P(y<y0)$; $P(x<x0)$, $P(y>y0)$; and $P(x>x0)$, $P(y>y0)$. The 2D KS test statistic is thus defined as the largest difference between empirical and theoretical distributions across each of these possible regions. In order to generate a test-statistic distribution under the null hypothesis, we performed a Monte-Carlo simulation where sets of random pairs of data points are sampled from the PDF formed by the product of the marginal distributions. The resulting bivariate CDF is compared to the theoretical CDF and the maximal difference is taken. This is performed repeatedly in order to generate a distribution of

maximal differences that would occur by chance. Finally, the test statistic is computed from the empirical distribution, and compared to this distribution at a 95% confidence level.

Movie acquisition system

Images were acquired on the IX81 inverted microscope system (Olympus) using the Metamorph acquisition software (Molecular Devices) with the iKon Charge Coupled Device (CCD) camera (Andor). Fluorophores were excited using X-Cite XLED1 light source (Lumen Dynamics) equipped with the BLX, BGX and GYX modules.

Movie data analysis: Segmentation and tracking

The Schnitzcells script package (Young et al., 2011) was used to segment and track cells from the acquired images. This package performs a number of procedures as described below. Briefly, cells were segmented with Matlab built-in edge detection script, using Laplacian of Gaussian method. Segmented cells in individual frames were then tracked across all time points by performing a point-matching algorithm on successive pairs of frames to generate a cell lineage data structure. To obtain the total fluorescence level of each cell, the images were "flattened" by correcting for the nonuniformity of illumination, followed by local background correction that takes into account the camera acquisition background, autofluorescence from the medium and fluorescence contribution from neighboring cells.

Movie data analysis: Production rate estimation and step detection

To enable the continuous estimation of production rates (slopes), frames around cell divisions are removed and fluorescence lost during divisions (to sister cells) is added back to the trace of interest to create a continuous total fluorescence trace for each lineage. Instantaneous fluorescence production rates were estimated by fitting the continuous total fluorescence of a 6-hour window to a linear section using the linear least squares method. Distributions of reporter production rates (Figs. 4A,B) were obtained by sampling the instantaneous fluorescence production rates of all cell lineages at 1-hour intervals. To

characterize abrupt changes in production rates, we identified sharp inflections of the continuous total fluorescence traces by applying a custom-built step detector on overlapping and consecutive 6-hour windows 15 minutes apart (Fig. S4D). For each window, we obtained fits to a linear polynomial model and a continuous two-piece linear polynomial model with a joint at midpoint using the linear least squares and non-linear least squares methods, respectively. The continuous two-piece linear model can be represented as follows:

$$y = \begin{cases} m_a x + c & , \quad x < x_{mid}, \\ m_a x_{mid} + c + m_b(x - x_{mid}) , & \quad x \geq x_{mid}, \end{cases}$$

where x_{mid} is the midpoint of the window.

We used two criteria to determine whether a given window fits better to the one-piece or two-piece linear fits: (1) whether the noisiness of the trace can explain the deviation of the data from the one-piece fit (mean sum of squared errors, M.S.E.), and (2) whether the two slopes obtained from the two-piece fit are significantly different from each other. For (1), we define the noisiness of the trace as the variance of the distribution of frame-to-frame fluctuations in total fluorescence, i.e. $var(Y_{t+1} - Y_t)$. For a perfectly linear trace without noise, the mean of $Y_{t+1} - Y_t$ equals the slope of the trace. As the observation noise increase, the SSE of one-piece fit increases even if the underlying trace has a constant slope. We therefore estimated the portion of SSE of one-piece fit unexplained by the noisiness of the trace as the residual noise, defined as $MSE_{1pc} / var(Y_{t+1} - Y_t)$, where *n* is the number of frames within a window. For (2) we obtained the 95% confidence bounds of the two slopes in the two-piece fit and determined if they overlap. Using (1) and (2), we identified stretches of frames where two-piece fit is significantly better than one-piece. The frame with the highest residual noise among each of these stretches was designated as the point of inflection and the rest of the trace was approximated by linear segments between these points.

Movie data analysis: Hidden Markov Model and Viterbi Algorithm

We set up a two-state HMM to estimate the frequency of state-switching events between the higher and

lower Nanog states. We assume each of the two states can produce an independent Gaussian distribution of production rates, with specified mean and variance, including potential overlap between the two states. Over each unit time, a cell can either stay at its current state or switch to the other state with specified probabilities. Thus, given a specific parameter set, there exists for the production rate time-series of each cell a corresponding series of underlying states that has the maximum likelihood. This likelihood is a balance between the probability of observing a production rate at the corresponding state and that of switching to another state, such that a cell that transiently exhibits a production rate far from the mean of its current state is more likely to be fluctuating rapidly within a state than switching away and back. The Baum-Welch algorithm (Do et al., 2008) maximizes the sum of this likelihood over all cells by iteratively changing the parameters in small increments, improving the total likelihood each time.

Prior to training the model with data, initial transition rates between the states in both directions were set at 0.0001/hour. Initial parameters for each state were set with the mean value drawn from the range of observed production rates and variance. Re-initializing the random parameters in the model yielded similar results. We employed the HMM toolbox for Matlab (Murphy, 1998), which generated maximum likelihood estimate of the model parameters using the Baum-Welch algorithm. Since the production rate sequences used to train the HMM contained repeated time-series when multiple lineages shared the same ancestor, the state-transition rates generated directly from HMM could be an overestimation. We applied the Viterbi algorithm (Rabiner, 1989) to combine the model parameter estimates and the observed data to infer the most likely state sequence for each cell lineage. From this we reported the empirical state-transition rates, normalized to the average length of a cell cycle.

Bursty transcription simulation and mixing time analysis

Bursty transcription was simulated using the model previously described (Peccoud and Ycart, 1995). In this model, a promoter can transit stochastically between an active and an inactive form. This is not to be confused with a cellular state, which is usually maintained over a longer timescale and within which a

gene bursts in a characteristic burst size and frequency. Transcription occurs only when the promoter is in its active form, producing a burst of mRNA molecules, which decay exponentially. To aid comparison between the simulated transcription dynamics and our experimental observations, we added protein production to the simulation. Further, since our fluorescence protein is stable, and to restrict the source of heterogeneity in our simulation to stochastic transcription, we assumed zero protein decay rate and deterministic protein production at a constant rate. Lastly, both mRNA and protein are partitioned when cells divide, which were set to have division rates similar to experimental data. This model can be described by the following reactions:

$$
\begin{aligned}
I &\xrightarrow{k_{on}} A \\
A &\xrightarrow{k_{off}} I \\
A &\xrightarrow{\beta_m} A + m \\
m &\xrightarrow{\alpha_m} \emptyset \\
m &\xrightarrow{\beta_p} m + p
\end{aligned}
$$

Here, A and I denote the promoter in its active and inactive forms, respectively; m -- mRNA level; p -- protein level; k_{on} and k_{off} -- activating and inactivating rates of the promoter, respectively; α_m -- mRNA degradation rate; β_m -- mRNA production rate; Ø -- mRNA degradation; β_p -- protein production rate.

A cellular state is thus characterized by the frequency of mRNA bursts and the mean number of mRNA molecules produced per bursts. Here, we considered one limiting case of this model, where k_{off} is significantly larger than k_{on} and somewhat larger than α_m. This assumption can be related physically to a scenario where bursts are relatively infrequent and have short durations, and the distribution of mRNA levels produced under these assumptions can be described with a single gamma (Raj et al., 2006) or NB function (Paulsson and Ehrenberg, 2000). A cell changes state in a gene when one or more of the parameters k_{on}, k_{off} or β_m for that gene is changed, thus resulting in different burst frequencies and sizes.

To simulate mRNA and protein dynamics for the *Nanog*-high state in serum+LIF condition (shown in

Figs. 4E, S4E), we used the following parameters estimated from mRNA distributions in smFISH: For *Nanog* -- burst size = 33 mRNA/hour, burst frequency = 0.39 bursts/hour; For *Oct4* -- burst size = 87 mRNA/hour, burst frequency = 0.52 bursts/hour. These assume that mRNA half-lives of *Nanog* and *Oct4* are 5.85 and 7.4 hours, respectively (Sharova et al., 2009).

We utilized computer simulations of this model to explore whether changes in state affect the intra-state dynamics of heterogeneity. Varying burst frequency and burst size results in traces with various frequencies of apparent steps when analyzed using the same step detector, which identified regimes in the bursty transcription parameter space where steps of similar quality to the ones observed can be generated (Figs. S4F, S5C). Furthermore, the resulting dynamics also display a wide range of shapes of fluctuation and levels of expression. We quantified these variations with an objective measurement, the "mixing time", a population metric adapted from Sigal et al. (2006). For each simulated population ($n = 200$ traces) using a single parameter set, we ranked all traces by their production rate at each time point. Thus a cell starting with the lowest production rate among the population may change in this rank when its production rate changes over time. We computed the autocorrelation function $A(\tau)$ of this rank for each population and the mixing time is defined as the time lag τ at which $A(\tau)$ decayed to 0.5. We opted to calculate the mixing time using production rate but not total fluorescence level because the stable fluorescent reporter facilitates accurate production rate estimate but may not reflect the physiological level of endogenous proteins. Additionally, for more direct comparison between the mixing times calculated from simulated and observed data, the production rates in simulation were computed using the simulated protein traces after Gaussian noise similar to the level observed was added.

Supplemental References

Do, C.B., and Batzoglou, S. (2008). What is the expectation maximization algorithm? Nature Biotechnology 26, 897–899.

Murphy, K.P. (1998). Hidden Markov Model (HMM) Toolbox for Matlab. Retrieved Dec 6, 2012, from http://www.cs.ubc.ca/~murphyk/Software/HMM/hmm.html.

Rabiner, L.R. (1989). A tutorial on hidden Markov models and selected applications in speech recognition. Proceedings of the IEEE *77*, 257–286.

Sharova, L., Sharov, A., Nedorezov, T., Piao, Y., Shaik, N., and Ko, M. (2009). Database for mRNA half-life of 19 977 genes obtained by DNA microarray analysis of pluripotent and differentiating mouse embryonic stem cells. DNA Research *16*, 45.

Young, J.W., Locke, J.C.W., Altinok, A., Rosenfeld, N., Bacarian, T., Swain, P.S., Mjolsness, E., and Elowitz, M.B. (2011). Measuring single-cell gene expression dynamics in bacteria using fluorescence time-lapse microscopy. Nature Protocols *7*, 80–88.
