# Supplementary figures and images for: Dynamic Heterogeneity and DNA Methylation in Embryonic Stem Cells
Source: Mol Cell. 2014 Jul 17;55(2):319–31. doi: 10.1016/j.molcel.2014.06.029 (PMC4104113; doi:10.1016/j.molcel.2014.06.029)

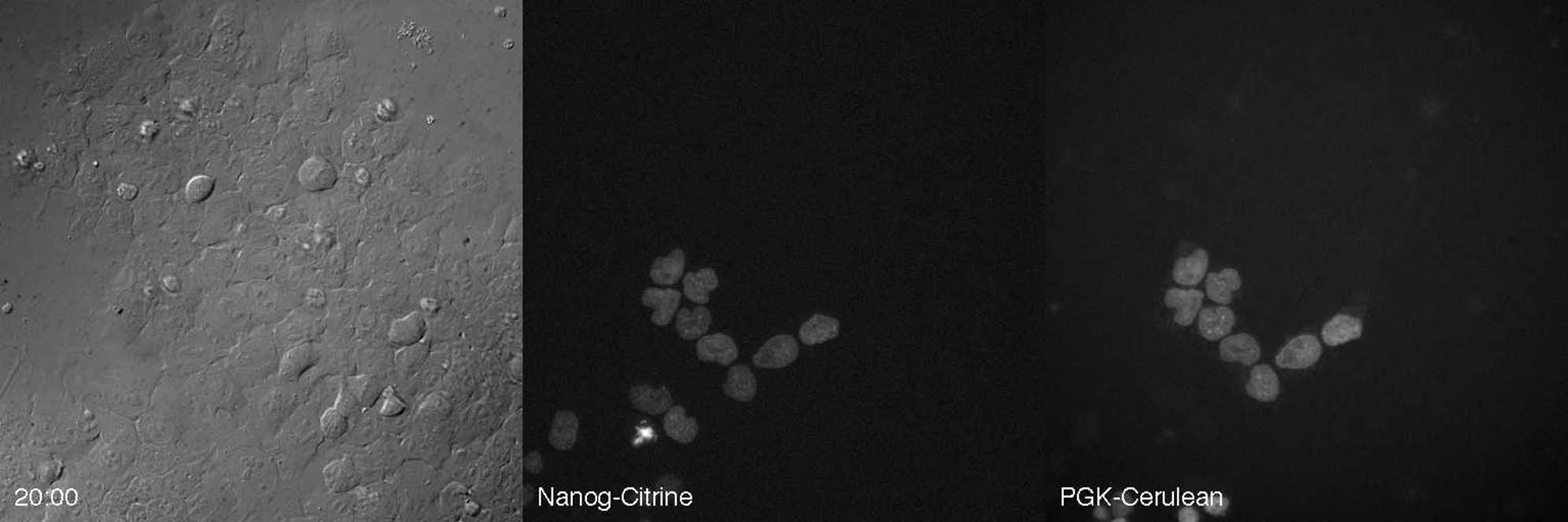

Supplement: Movie S1. Nanog-High to Nanog-Low Switch in Serum + LIF — Cells imaged in serum + LIF condition. Shown are examples of cells switching from Nanog-high to Nanog-low. Related to Figure 4. [file mmc3.jpg]

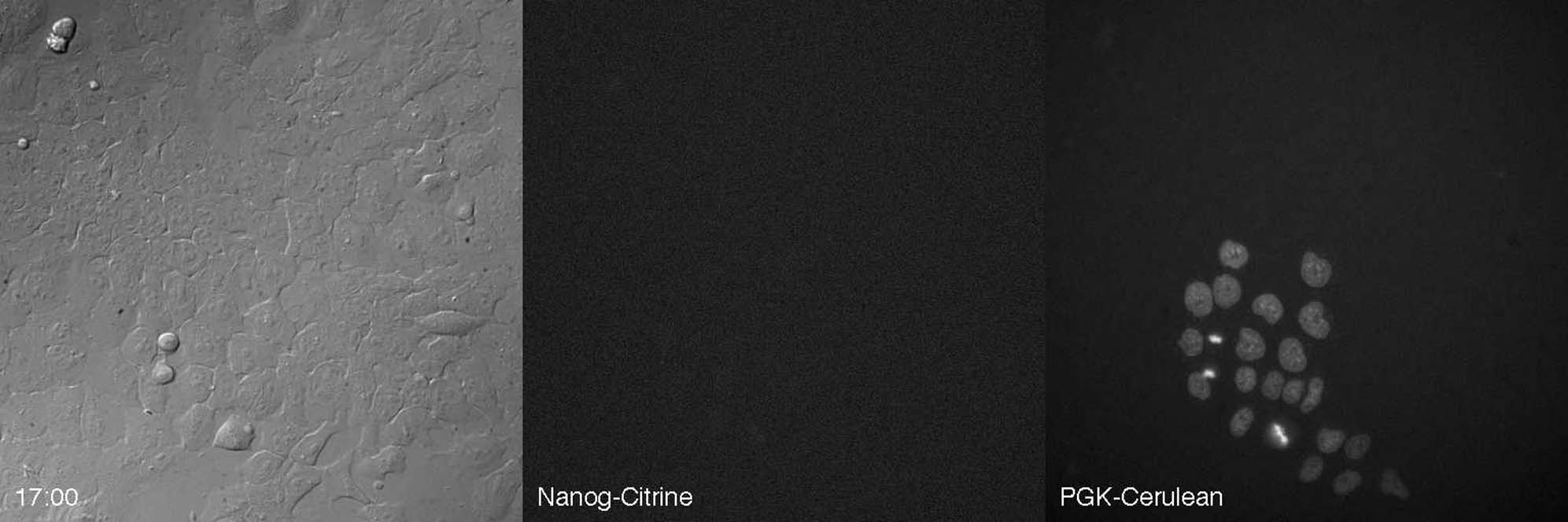

Supplement: Movie S2. Nanog-Low to Nanog-High Switch in Serum + LIF — Cells imaged in serum + LIF condition. One of the lineages switched from Nanog-low to Nanog-high. Related to Figure 4. [file mmc4.jpg]

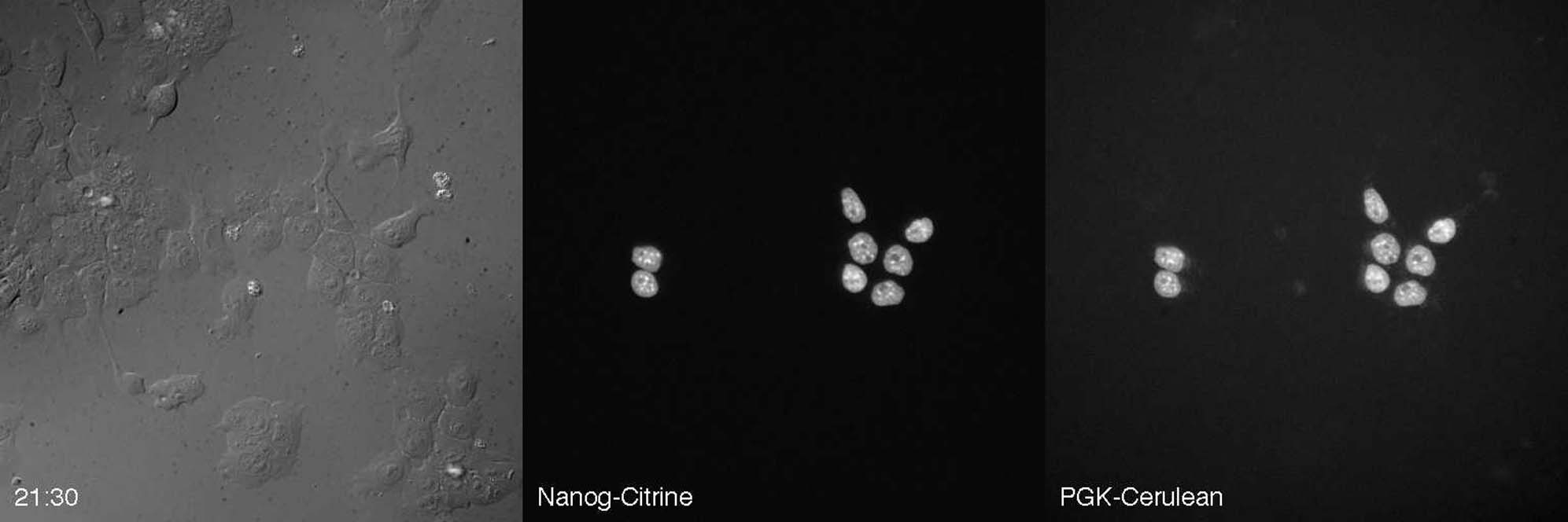

Supplement: Movie S3. Nanog-SH Cells in 2i + Serum + LIF — Cells imaged in 2i + serum + LIF condition. Nanog reporter expression is homogeneous compared to cells grown without 2i. Related to Figure 5. [file mmc5.jpg]

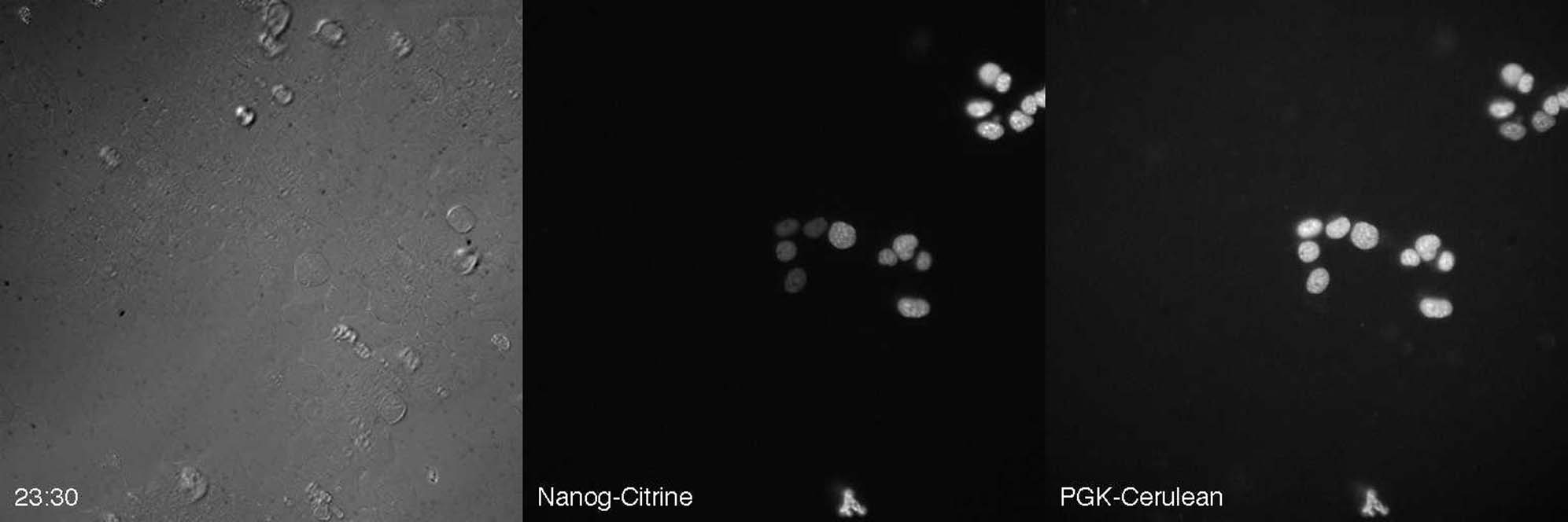

Supplement: Movie S4. Nanog-Low to Nanog-SH Switch in 2i + Serum + LIF — Cells imaged in 2i + serum + LIF condition. Shown are Nanog-low cells, which were rare and switched to Nanog-SH. Related to Figure 5. [file mmc6.jpg]
