# Supplementary material for: Dynamic Heterogeneity and DNA Methylation in Embryonic Stem Cells
Source: Mol Cell. 2014 Jul 17;55(2):319–31. doi: 10.1016/j.molcel.2014.06.029 (PMC4104113; doi:10.1016/j.molcel.2014.06.029)
Supplement: Document S2. Article plus Supplemental Information [file mmc7.pdf]

Molecular Cell
Resource

CellPress

Dynamic Heterogeneity and DNA Methylation in Embryonic Stem Cells

Zakary S. Singer,[1,7] John Yong,[2,7] Julia Tischler,[6] Jamie A. Hackett,[6] Alphan Altinok,[2,3] M. Azim Surani,[6] Long Cai,[4] and Michael B. Elowitz[5,*]
[1]Computation and Neural Systems
[2]Division of Biology
[3]Biological Network Modeling Center
[4]Program in Biochemistry and Molecular Biophysics and Division of Chemistry and Chemical Engineering
[5]Howard Hughes Medical Institute and Division of Biology and Department of Applied Physics
California Institute of Technology, Pasadena, CA 91125, USA
[6]The Wellcome Trust/Cancer Research UK Gurdon Institute, The Henry Wellcome Building of Cancer and Developmental Biology, University of Cambridge, Tennis Court Road, Cambridge CB2 1QN, UK
[7]Co-first author
*Correspondence: melowitz@caltech.edu
http://dx.doi.org/10.1016/j.molcel.2014.06.029

SUMMARY

Cell populations can be strikingly heterogeneous, composed of multiple cellular states, each exhibiting stochastic noise in its gene expression. A major challenge is to disentangle these two types of variability and to understand the dynamic processes and mechanisms that control them. Embryonic stem cells (ESCs) provide an ideal model system to address this issue because they exhibit heterogeneous and dynamic expression of functionally important regulatory factors. We analyzed gene expression in individual ESCs using single-molecule RNA-FISH and quantitative time-lapse movies. These data discriminated stochastic switching between two coherent (correlated) gene expression states and burst-like transcriptional noise. We further showed that the "2i" signaling pathway inhibitors modulate both types of variation. Finally, we found that DNA methylation plays a key role in maintaining these metastable states. Together, these results show how ESC gene expression states and dynamics arise from a combination of intrinsic noise, coherent cellular states, and epigenetic regulation.

INTRODUCTION

Many cell populations appear to consist of mixtures of cells in distinct cellular states. In fact, interconversion between states has been shown to underlie processes ranging from adult stem cell niche control (Lander et al., 2009; Rompolas et al., 2013) to bacterial fitness (Süel et al., 2006) to cancer development (Gupta et al., 2011). A central challenge is to identify transcriptional states, along with the mechanisms that control their stability and generate transitions among them.

Single-cell transcriptional studies have revealed substantial gene expression heterogeneity in stem cells (Canham et al., 2010; Chambers et al., 2007; Chang et al., 2008; Guo et al., 2010; Yamanaka et al., 2010). Moreover, subpopulations expressing different levels of *Nanog*, *Rex1*, *Dppa3*, or *Prdm14* show functional biases in their differentiation propensity (Hayashi et al., 2008; Singh et al., 2007; Toyooka et al., 2008; Yamaji et al., 2013). This heterogeneity could in principle arise from stochastic fluctuations, or "noise," in gene expression (Eldar and Elowitz, 2010; Raj et al., 2008; Zenklusen et al., 2008). Alternatively, it could reflect the coexistence of multiple cellular states, each with a distinct gene expression pattern showing correlation between a set of genes (Guo et al., 2010; Gupta et al., 2011; Jaitin et al., 2014; Shalek et al., 2013). Disentangling these two sources of variation is important for interpreting the transcriptional states of individual cells and understanding stem cell dynamics.

A related challenge is to understand the mechanisms that stabilize cellular states despite noise. DNA methylation has been shown to be heritable over many generations, is critical for normal development (Okano et al., 1999), and may help stabilize irreversible cell fate transitions (Hackett et al., 2013; Reik, 2007; Schübeler et al., 2000; Smith et al., 2012). However, the role of DNA methylation in the reversible cell state transitions that underlie equilibrium population heterogeneity has been much less studied (Fouse et al., 2008; Mohn et al., 2008). Recently, it was reported that exposing ESCs to inhibitors of MEK and GSK3β (called 2i) abolishes heterogeneity and induces a "naïve" pluripotent state (Marks et al., 2012; Wray et al., 2011) with reduced methylation (Ficz et al., 2013; Habibi et al., 2013; Leitch et al., 2013). However, a causal role linking methylation, heterogeneity, and 2i remains to be elucidated.

Together, these observations provoke several fundamental questions: First, how do noise and states together determine the distribution of expression levels of individual regulatory genes (Figure 1A)? Second, how do gene expression levels vary dynamically in individual cells, both within a state and during transitions between states (Figure 1B)? Finally, how do cells stabilize metastable gene expression states, and what role does DNA methylation play in this process?

CrossMark

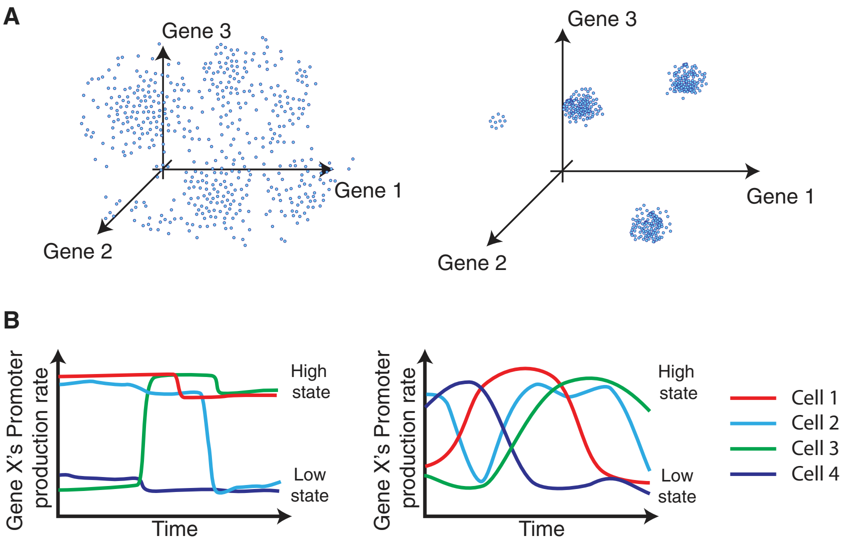

Figure 1. Different Types of Gene Expression Heterogeneity

(A) Intrinsic noise in gene expression can lead to uncorrelated variation (left), while the coexistence of distinct cellular states can produce correlated variability in gene expression (right). Both panels depict schematic static "snapshots" of gene expression.

(B) Dynamically, gene expression levels could vary infrequently and abruptly (left) or more frequently and gradually (right) both within and between cellular states (schematic).

Using single-molecule RNA-FISH (smFISH), we analyzed the structure of heterogeneity in the expression of key cell fate regulators, finding that distinct cell states account for most variation in some genes, while others are dominated by stochastic bursts. Using time-lapse movies of individual cells, we observed abrupt, step-like dynamics due to cell state transitions and transcriptional bursts. Finally, using perturbations, we observed that DNA methylation modulates the population fraction of cells in the two states, consistent with reciprocal expression of the methyltransferase *Dnmt3b* and the hydroxymethylase *Tet1*. Together, these results suggest how noise, dynamics, and epigenetic regulatory mechanisms contribute together to the overall distribution of gene expression states in stem cell populations.

RESULTS

Mouse ESCs Show Three Distinct Types of Gene Expression Distributions

The process of mRNA transcription is inherently stochastic. As a result, even a clonal cell population in a single state is expected to display variability in the copy number of each mRNA (Blake et al., 2003; Elowitz et al., 2002; Friedman et al., 2006; Ozbudak et al., 2002; Paulsson and Ehrenberg, 2000; Peccoud and Ycart, 1995; Raj et al., 2006; Shahrezaei and Swain, 2008; Suter et al., 2011), potentially leading to phenotypic differences between otherwise identical cells (Cai et al., 2006; Choi et al., 2008; Maamar et al., 2007; Süel et al., 2006; Zong et al., 2010).

In order to accurately measure mRNA copy numbers in large numbers of individual ESCs, we developed an automated platform for smFISH (Supplemental Information). This system enables rapid analysis of four genes per cell across ~400 cells per sample (Figures S1A–S1D). We validated the system by comparing three measures of expression of the same gene in the same cells using a Rex1-dGFP reporter line (Wray et al., 2010) (Figure S1E).

Using this platform, we analyzed 36 pluripotency-associated regulators that play critical roles in ESCs or are heterogeneously expressed, as well as several markers of early cell fates and housekeeping genes. The resulting mRNA distributions exhibited a range of distribution shapes and degrees of heterogeneity (Figure 2A). We analyzed these distributions within the framework of bursty transcriptional dynamics. In this model, mRNA production occurs in stochastic bursts that are brief compared to the mean interburst interval and are exponentially distributed in size. Bursty dynamics produce negative binomial (NB) mRNA distributions (Paulsson et al., 2000; Raj et al., 2006), whose shape is determined by the frequency and mean size of bursts.

Genes exhibited three qualitatively distinct types of mRNA distributions. First, most genes were unimodal and well-fit by a single NB distribution (Figures 2B and S2A, maximum likelihood estimation [MLE], χ^2 goodness of fit [GOF] test $p > 0.05$). This class included *Oct4*, *Rest*, *Tcf3*, *Smarcc1*, *Sall4*, and *Zfp281*. Coefficients of variation (CV) were typically ~0.5 for the most homogeneous genes (Figure 2A).

Second, a subset of unimodal genes exhibited "long-tailed" distributions, in which most cells had few, if any, transcripts, while a small number of cells displayed many transcripts. These distributions were also well fit by a single NB distribution, but with resulting distributions that generally decreased monotonically with increasing mRNA concentration (Figures 2B and S2A, χ^2 GOF $p > 0.05$). The most heterogeneous long-tailed genes had burst frequencies of less than one burst per mRNA half-life. These included *Tbx3* (CV = 2.13 ± 0.23, mean ± SEM), *Dppa3* (CV = 1.76 ± 0.31), and *Prdm14* (CV = 1.599 ± 0.20). Other long-tailed genes such as *Pecam1*, *Klf4*, *Blimp1*, *Socs3*, *Nr0b1*, and *Fgfr2* had higher burst frequencies and less skew. Long-tailed genes arising from rare bursts could provide a source of stochastic variation that could propagate to downstream genes.

Third, there were some genes whose mRNA distributions were significantly better fit by a linear combination of two NB distributions than by one (Supplemental Information, Akaike's Information Criteria [AIC] and log-likelihood ratio test, $p < 0.05$). These genes included *Rex1*, *Nanog*, *Esrrb*, *Tet1*, *Fgf4*, *Sox2*, *Tcl1*, and *Lifr* (Figures 2B and S2A). In some cases, the two components of these distributions were well separated from one another (e.g., *Rex1* and *Esrrb*), while in other cases they overlapped strongly (e.g., *Nanog* and *Lifr*), such that the absolute number of transcripts for a single gene did not accurately indicate to

which state the cell belonged. These bimodal distributions suggested the existence of multiple cell states (see below).

Markers of most differentiated fates including *Pax6* (neuroectoderm), *Fgf5* (epiblast), *Sox17* and *FoxA2* (definitive endoderm), and *Gata6* (primitive endoderm) showed no detectable expression (data not shown). However, the mesendodermal regulator *Brachyury* (*T*) was expressed at a level of ~5–20 transcripts in 6% of *Rex1*-low cells. Similarly, the two-cell-like state marker *Zscan4c* (Macfarlan et al., 2012) showed ~3–60 transcripts in 3% of cells (Figure S2A). These genes did not fit well to NB distributions, suggesting that processes other than transcriptional bursting impact their expression in this small fraction of cells.

Bimodal Genes Vary Coherently

We next used the smFISH data to determine whether the bimodal genes were correlated, which would suggest their control by a single pair of distinct cell states, or varied independently, which would suggest a multiplicity of states. The data revealed a cluster of bimodal genes that correlated with one another. *Rex1*, *Nanog*, and *Esrrb* displayed the strongest correlations ($r = \sim 0.7$, Figures 2C and S2B), while genes with strong overlap between modes, such as *Tcl1*, *Lifr*, *Sox2*, and *Tet1*, displayed somewhat weaker, but still significant, correlations ($r = \sim 0.5$, Figures 2C and S2B), beyond those observed between bimodal and nonbimodal genes (e.g., $r = 0.2$ for *Rex1* and *Oct4*). Thus, a cell in the high- or low-expression state of one bimodal gene is likely to be in the corresponding expression state of others. Some correlations were negative: expression of the de novo methyltransferase *Dnmt3b* was reduced in the *Rex1*-high state ($r = -0.46$, Figure 2C). Note that cell cycle effects did not explain these correlations (Figure S2C). Together, these data suggest that bimodal genes appear to be broadly coregulated in two distinct states.

Long-tailed genes exhibited more complex relationships. Those with very large variation (CV > 1.5) were correlated with the expression state of the bimodal gene cluster, but not with one another (Figures 2C, 2D, and S2B). For example, genes like *Dppa3*, *Tbx3*, and *Prdm14* burst predominantly in the *Rex1*-high state, but even in this state, most cells showed no transcripts of these genes ($p < 0.001$, see Supplemental Information for statistical analysis). Thus, it appears that these genes are expressed in infrequent, stochastic bursts that occur mainly in one of the two cellular states.

Interestingly, expression of *Socs3*, a negative regulator and direct target of Stat signaling (Auernhammer et al., 1999), appeared conditional on expression of its bimodally expressed receptor *Lifr* (note absence of *Socs3* expression in low-*Lifr* cells in Figure S2B). Analysis of additional regulators not measured here could in principle reveal additional states or more complex distributions. Overall, however, the multidimensional mRNA distributions measured here are consistent with a simple picture based on two primary states and stochastic bursting.

The Two Primary States Exhibit Distinct DNA Methylation Profiles

These data contained an intriguing relationship between three factors involved in DNA methylation: the de novo methyltransferase *Dnmt3b*, the hydroxylase *Tet1*, which has been implicated in removing methylation (Ficz et al., 2011; Ito et al., 2010; Koh et al., 2011; Wu et al., 2011), and *Prdm14*, which represses expression of *Dnmt3b* (Grabole et al., 2013; Leitch et al., 2013; Ma et al., 2011; Yamaji et al., 2013). While *Rex1* was anticorrelated with *Dnmt3b* expression and positively correlated with *Tet1* (Figure 3A), *Prdm14* showed a long-tailed distribution conditioned on the *Rex1*-high state (Figure 2D). Based on these relationships and the observation that methylation increases during early development (Meissner et al., 2008), we hypothesized that the *Rex1*-low state might exhibit increased methylation compared to the *Rex1*-high state.

To test this hypothesis, we sorted *Rex1*-high and -low cells using the Rex1-dGFP reporter line and performed locus-specific bisulfite sequencing at known targets of methylation *Dazl*, *Mael*, and *Sycp3* (Borgel et al., 2010) (Figures 3B, S3A, and S3B). These promoters exhibited 2–3 times greater methylation in *Rex1*-low cells compared to *Rex1*-high cells, indicating the two states are differentially methylated in at least some genes. In contrast, *Rex1*-low cells that subsequently reverted to the *Rex1*-high state recovered the methylation levels of *Rex1*-high cells, indicating that methylation is reversible. Similarly, quantitative ELISA analysis demonstrated both differential methylation and reversibility in global methylation levels (Figure 3C).

We next asked more generally which genes exhibited differential promoter methylation. We again sorted *Rex1*-high and -low cells and assayed DNA methylation by reduced representation bisulfite sequencing (RRBS), analyzing regions 2 kb upstream to 500 bp downstream of each ESC-expressed mRNA transcriptional start site (Meissner et al., 2008; Marks et al., 2012). The distributions of methylation levels across genes were bimodal in both *Rex1* states, with the more highly methylated peak shifted to even higher methylation levels in *Rex1*-low cells (Figure 3D). By analyzing the shift in methylation on a gene-by-gene basis, we found that the increase in methylation in *Rex1*-low cells occurred predominantly through increased methylation of the promoters that were more highly methylated in *Rex1*-high cells (Figures 3E and S3C). Thus, the change in promoter methylation occurs in a specific subset of promoters. Furthermore, the overall methylation level of a gene was related to the number of CpGs in its promoter, such that the larger the CpG content of a promoter, the lower its methylation in both states. Not all gene promoters were well covered by RRBS. However, among those that were, several key ESC regulators including *Esrrb*, *Tet1*, and *Tcl1* all showed increased levels of methylation in the *Rex1*-low state. Figure 3E (inset) and Figure S3C show methylation levels of individual CpGs. These results provide a view of the change in promoter methylation that occurs during transitions between the *Rex1*-high and -low states.

Bursty Transcription Generates Dynamic Fluctuations in Individual Cells

Evidently, cells populate two transcriptional states, each characterized by distinct methylation profiles. To understand the dynamic changes in gene expression that occur as individual cells switch between these states, we turned to time-lapse microscopy. We analyzed transcriptional reporter cell lines for *Nanog* and *Oct4*, each containing a histone 2B (H2B)-tagged fluorophore expressed under the control of the corresponding

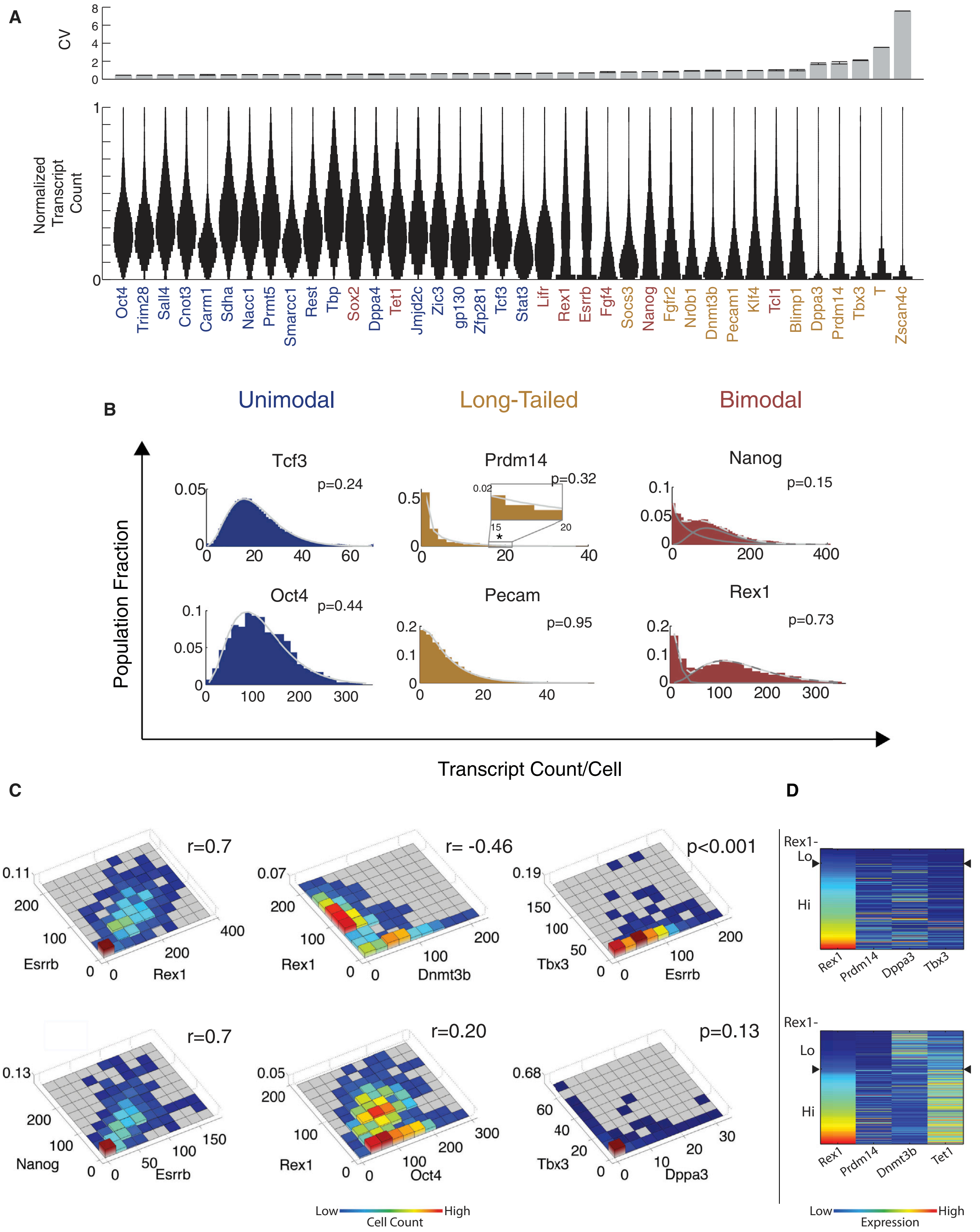

(legend on next page)

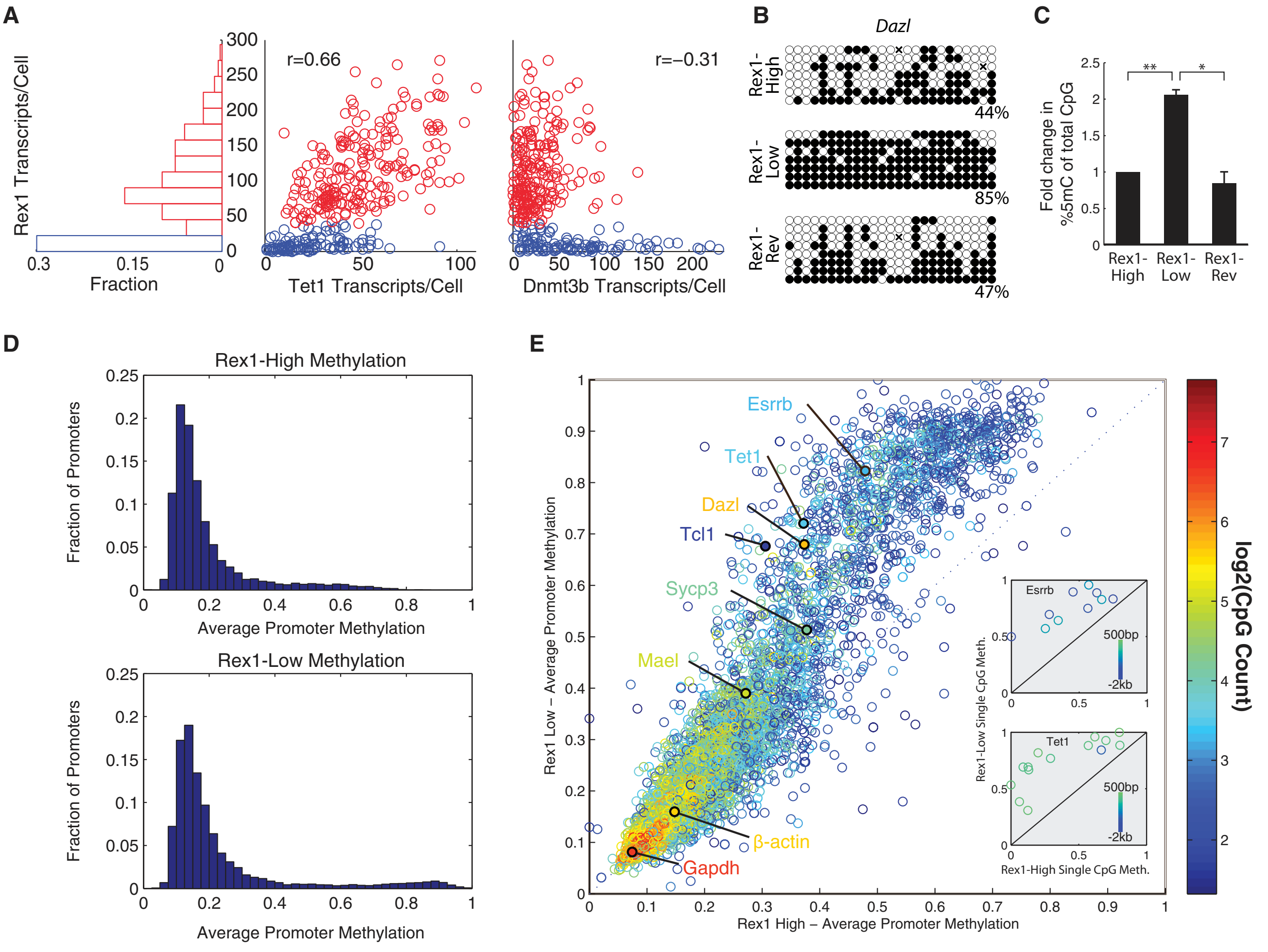

Figure 3. The Two *Rex1* States Are Differentially Methylated

(A) smFISH measurements show that *Rex1* bimodality is correlated with *Tet1* and anticorrelated with *Dnmt3b* expression.

(B) Locus-specific bisulfite sequencing of the *Dazl* promoter. Methylation levels shown are in the *Rex1*-high (top), *Rex1*-low (middle), and *Rex1*-low-to-high reverting (bottom) populations.

(C) Global levels of 5mC measured by quantitative ELISA in the *Rex1*-high, -low, and -low-to-high reverting cells. Data shown are mean ± SD from two independent experiments. *, $p < 0.05$; **, $p < 0.01$; by two-sample t test.

(D) Histogram of promoter methylation shows bimodality in the *Rex1*-high (top) and -low (bottom) states, as quantified by RRBS.

(E) Scatter plot of promoter methylation between *Rex1*-high and -low states. Each point is the methylation fraction of a single gene promoter, color-coded by the number of CpGs in that promoter. Divergence from the diagonal implies differential methylation between states. Inset: Single CpGs in the promoter of the specific gene labeled, color coded by distance from TSS; see Figure S3C for additional genes.

promoter (Figures S4A and S4B; see also Supplemental Information). We imaged the reporter cell lines for ~50 hr periods with 15 min intervals between frames and segmented and tracked individual cells over time in the resulting image sequences. For each cell lineage, we quantified the instantaneous reporter production rate, defined as the rate of increase of total fluorescent protein in the cell, corrected for the partitioning of fluorescent protein into daughter cells during cell division (Supplemental Information). The H2B-fluorescent protein degradation rate is negligible under these conditions (Figure S4C), enabling us to

Figure 2. smFISH Reveals Gene Expression Heterogeneity and Correlation

(A) Top: coefficients of variation (CV, mean ± SEM) for ESC-associated regulators and housekeeping genes. Bottom: Distributions (violin plots) normalized by maximum expression level reveal qualitatively distinct gene expression distributions. Genes are sorted by increasing CV.

(B) Smoothed histograms for mRNA distributions overlaid with NB fits. Solid lines show individual NB distributions. Dashed gray lines show their sum (for bimodal genes). * denotes 95th percentile for *Prdm14*. P value: χ^2 goodness of fit test.

(C) Pairwise relationships between genes, analyzed by smFISH (r, Pearson correlation coefficient; p value by 2D K-S test (see Experimental Procedures and Figures S2A and S2B).

(D) Heat maps show examples of four-dimensional data sets.

use the reporter production rate as a measure of instantaneous mRNA level. An advantage of this approach is that it provides relatively strong fluorescence signals per cell, but still enables high time-resolution analysis (Locke and Elowitz, 2009). Consistent with static smFISH distributions, the production rate distributions of the *Nanog* and *Oct4* fluorescent reporters were bimodal and unimodal, respectively (Figure 4A).

Dynamically, cells remained in either one of two distinct *Nanog* expression states for multiple cell cycles (Figure 4B). During these periods, expression levels varied over the full range of Nanog expression levels within each state, with no evidence for persistent substates. However, closer examination revealed fluctuations within a single state, which typically occurred in discrete steps separated by periods of steady expression (Figure 4C). Using a computational step detection algorithm (Figure S4D and Supplemental Information), we found that *Nanog* and *Oct4* reporters exhibited 0.38 and 0.29 steps per cell cycle, respectively. These steps occurred in a cell cycle phase-dependent manner (Figure 4D), with down-steps clustered around cell division events and up-steps more broadly distributed across cell cycle phases.

Could these step-like dynamics arise simply from transcriptional bursting? To address this question, we simulated single-cell mRNA and protein traces using the bursty transcription model, with parameters determined from the NB fits of the static mRNA distributions (Supplemental Information). These simulations generated dynamic traces resembling those observed experimentally (Figures 4E and S4E). In the simulations, mRNA half-life and burst frequency determine the characteristics of detectable steps (Figure S4F); in general, steps were most prominent at low burst frequencies and short mRNA half-lives and became difficult to discriminate at high burst frequencies and long mRNA half-lives.

Step-like dynamics appear to be a natural consequence of stochastic expression, with up-steps reflecting burst-like production of mRNA and down-steps resulting from ~2-fold reduction in mRNA copy number at cell division (Figure S4G). This interpretation is consistent with the observed clustering of down-steps around cell division events and a more uniform cell cycle distribution of up-steps (Figure 4D). Because large bursts can effectively cancel out mRNA dilution at cell division events, they may appear underrepresented near cell division events. Note that most cell cycles showed no up-steps, suggesting that they are not due to increased gene dosage after chromosome replication (Brewster et al., 2014; Rosenfeld et al., 2005).

Dynamic Transitions between Cellular States

We next asked how cells transition dynamically between states. Previous work has relied on cell sorting, which can distort the signaling environments. By contrast, movies enable direct observation of switching events within a mixed cell population. Since the *Nanog* reporter production rate fluctuates even within a single state, we used a hidden Markov model (HMM) to classify each cell as either *Nanog*-high or *Nanog*-low at every point in time (Supplemental Information). We trained the HMM using time-series data of *Nanog* reporter production rates, sampled at fixed intervals across all tracked cell cycles, and used it to identify switching events and estimate switching frequencies.

Transitions from the *Nanog*-low to the *Nanog*-high state or vice versa occurred at a rate of 2.3 ± 0.25, or 7.9 ± 1.2, transitions per 100 cell cycles (mean ± SD), respectively (Figures 4F and 4G). These events did not correlate between sister cells (Table S1), consistent with independent, stochastic events. Interstate switching on average showed bigger and longer-lasting fold changes than intrastate steps in production rates (Figure S4H). Together, these results suggest that gene expression dynamics are dominated by a combination of step-like changes due to bursty transcription on shorter timescales and abrupt, apparently stochastic interstate switching events on longer timescales.

"2i" Inhibitors Modulate Bursty Transcription and State-Switching Dynamics

We next asked how gene expression heterogeneity and dynamics change in response to key perturbations. Dual inhibition of MEK and GSK3β, known as "2i," were shown to enhance pluripotency and reduce *Rex1* and *Nanog* heterogeneity (Marks et al., 2012; Wray et al., 2010). However, it has remained unclear how 2i affects the distribution of other heterogeneously expressed regulatory genes and what impact it has on dynamic fluctuations in gene expression.

We found that addition of 2i to serum + LIF media reduced variability in the mRNA levels of most genes (Figure 5A). In principle, this could reflect the elimination of one cellular state and/or changes in burst parameters. In 2i, the bimodal genes from Figure 2A became unimodal, suggesting that 2i suppresses one of the two cellular states (Figures 5A and S5A). In the case of *Nanog*, the remaining state increased its mean transcript level by ~1.5-fold, to what we term *Nanog*-SH (super high). *Tet1*, *Sox2*, and *Tcl1* also became unimodal, but displayed an overall decrease in absolute expression. With long-tailed genes, we found that mean *Dppa3* expression decreased slightly, while *Prdm14* and *Tbx3* became more homogenously expressed, exhibiting an increase in mean expression by ~300% and ~1,000%, respectively. These changes reflect the fact that nearly all cells were now observed to express *Prdm14* and *Tbx3*. Thus, 2i appeared to reduce variability in most genes, either by eliminating bimodality or by increasing their burst frequency.

Recently, it was shown that 2i-treated cells exhibit differentiation propensity similar to sorted *Rex1*-high subpopulations in embryoid body formation, suggesting they may represent similar functional states (Marks et al., 2012). We used the time-lapse movie system to compare the dynamic behavior of 2i-treated cells to that of cells in the *Rex1*-high subpopulation. Consistent with mRNA measurements, 2i shifted most cells into a *Nanog*-SH state (96% of total), characterized by ~3-fold higher median production rate compared to the *Nanog*-high state in serum + LIF (Figure 5B). Only a small fraction of cells showed expression overlapping with the *Nanog*-low state in serum + LIF at the beginning of the movies (after 6 days in treatment). Moreover, these cells switched to the *Nanog*-SH state at a >40-fold higher rate than the *Nanog*-low to *Nanog*-high switching rate measured in serum + LIF, with no reverse transitions observed (Figures 5C and S5B). These observations suggest that 2i increases the *Nanog*-low to *Nanog*-high

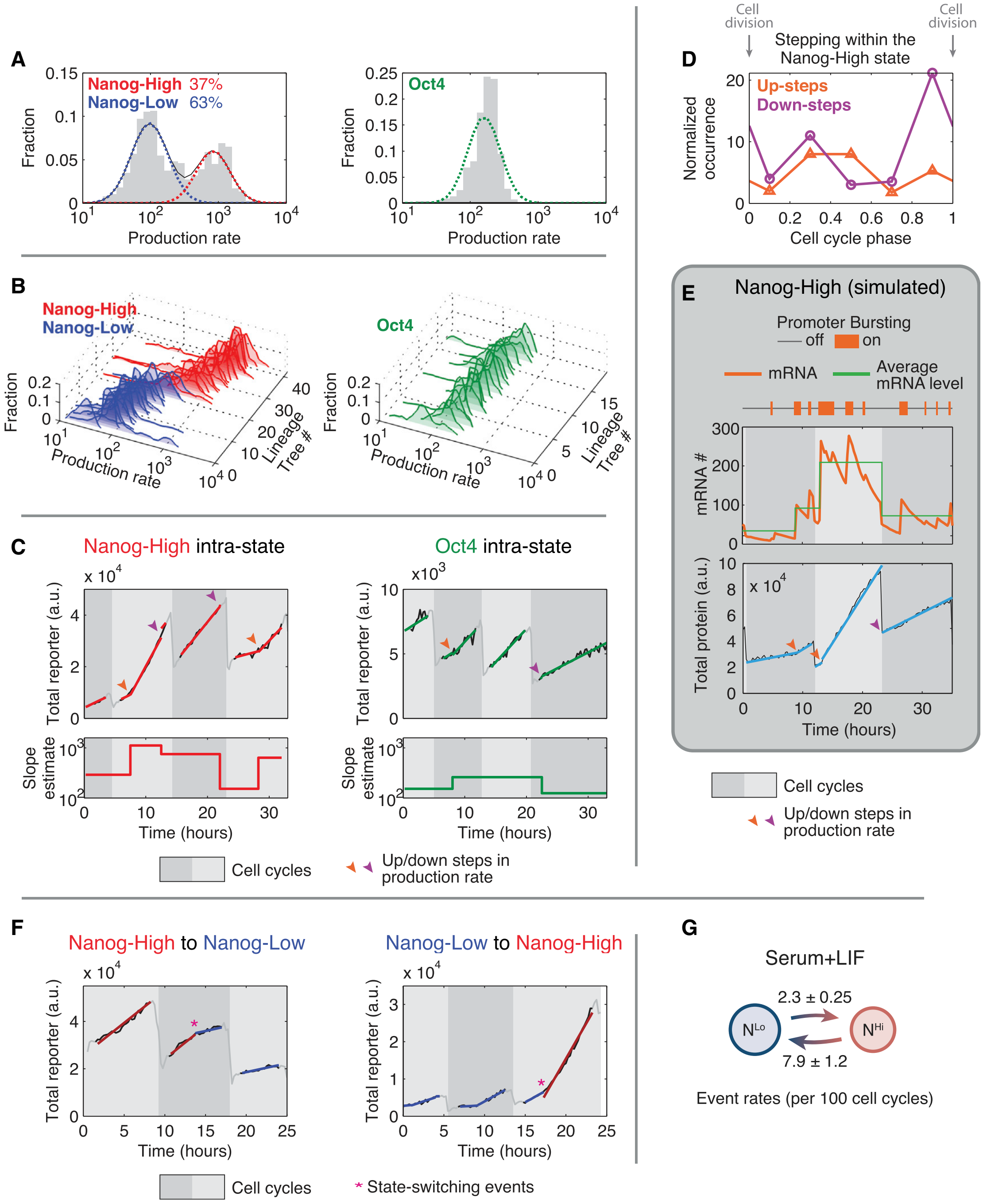

Figure 4. Movies Reveal Transcriptional Bursting and State-Switching Dynamics in Individual Cells

(A) Distribution of *Nanog* and *Oct4* production rates from representative movies in serum + LIF, and Gaussian fits to the components. Production rates were extracted from a total of 376 and 103 tracked cell cycles for *Nanog* and *Oct4*, respectively.

(B) Production rate distributions of individual cell lineage trees, each consisting of closely related cells descending from a single cell. Lineage trees are color-coded by the state they spend the majority of time in.

(C) Example single lineage traces exhibiting step-like changes in production rates within a state.

(D) Cell cycle phase distribution of steps within the *Nanog*-high state. Step occurrences are normalized by the frequencies of each cell cycle phase observed in the tracked data.

(E) Representative trace showing apparent steps from simulations under the bursty transcription model, using parameters estimated from mRNA distribution for the *Nanog*-high state (see Supplemental Information; see Figure S4E for simulation of *Oct4* dynamics).

(F) Example traces of individual cells switching between *Nanog*-low and *Nanog*-high states.

(G) Empirical transition rates (mean ± SD) between the two *Nanog* states (N^{Hi}, *Nanog*-high; N^{Lo}, *Nanog*-low).

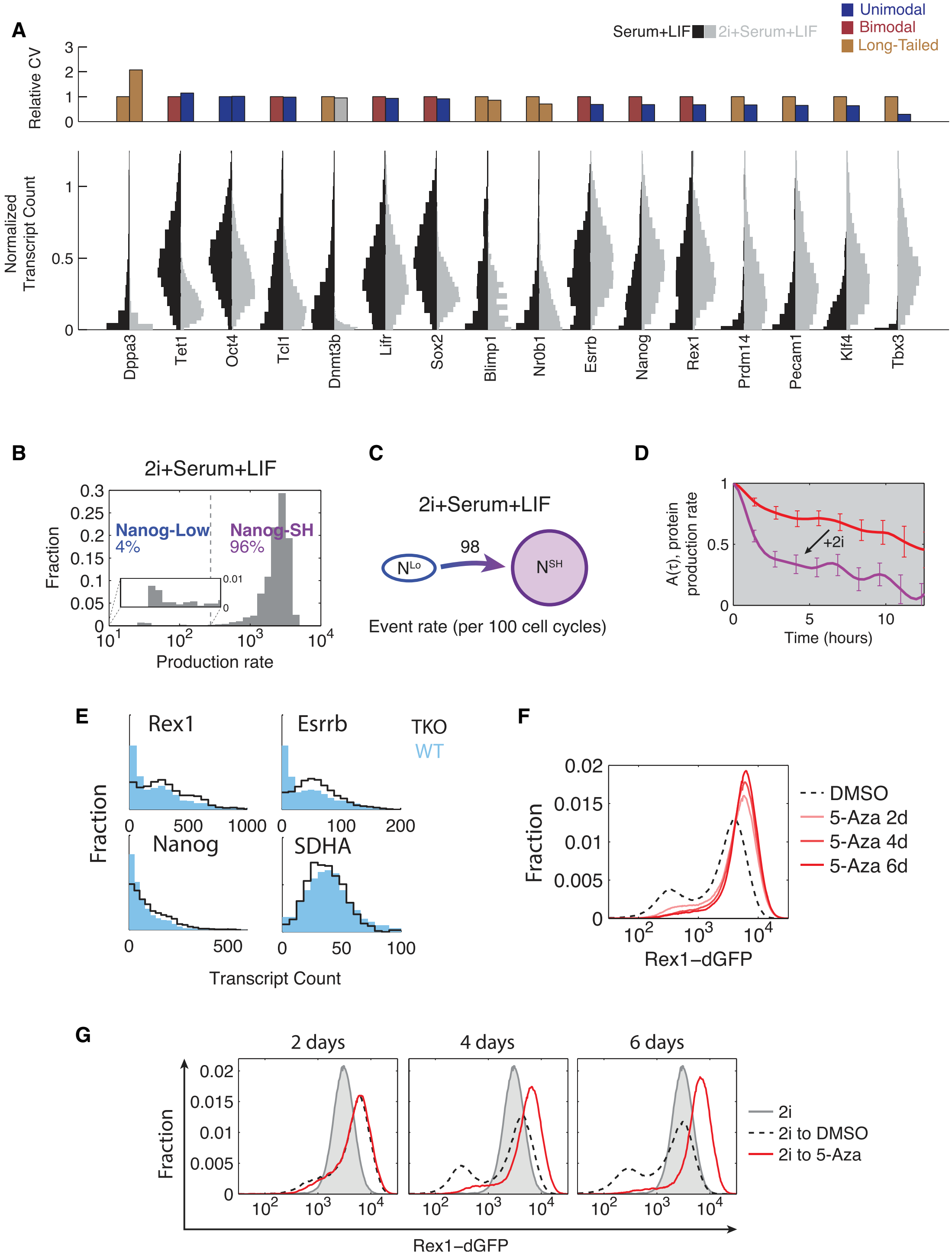

Figure 5. 2i and DNA Methylation Modulate Bursty Transcription and State-Switching Dynamics

(A) Comparison of mRNA distributions and CV between cells grown in serum + LIF and 2i + serum + LIF. Top: For each gene, the CV in serum + LIF is plotted on the left, and the CV for 2i + serum + LIF is plotted on the right. *Dnmt3b* in 2i + serum + LIF is represented in gray to reflect its marginal case of poor quality of fit in both bimodal and long-tailed models. Bottom: The left half of each violin represents the mRNA distribution in serum + LIF, while the right represents 2i + serum + LIF. Each gene's distributions are normalized by a value corresponding to the larger 95th percentile between the two treatments.

(B) Distribution of *Nanog* production rates from movies in 2i + serum + LIF.

(C) Empirical transition rates between the two *Nanog* states in the presence of 2i (N^{Lo}, *Nanog*-low; N^{SH}, *Nanog*-SH).

(legend continued on next page)

switching rate and reduces or eliminates *Nanog*-high to *Nanog*-low transitions (Figure 5C).

What effect, if any, does 2i have on the dynamics of gene expression noise? Static distributions suggested that 2i increased *Nanog* burst frequency ~45% from 0.39 to 0.55 burst/hr using the *Nanog* mRNA half-life previously estimated (Table S1 in Sharova et al., 2009) and assuming no change between conditions. To analyze the effects on dynamics, we computed the "mixing time," previously introduced to quantify the mean timescale over which a cell maintains a given expression level relative to the rest of the cell population (Sigal et al., 2006). Simulations of the bursty gene expression model showed that higher burst frequencies lead to faster mixing times, while burst size has little effect (Figure S5C). Together with smFISH measurements, this model predicted that Nanog mixing times should be faster in 2i. Qualitatively consistent with this prediction, the mixing time of *Nanog* production rate was reduced from 8.5 hr in *Nanog*-high in serum + LIF media to 1.7 in *Nanog*-SH cells in 2i-containing media (Figures 5D and S5D).

Together, these results indicate that 2i impacts ESC heterogeneity at three levels: First, it reduces gene expression variation in many, but not all, genes. Second, it eliminates one cell state by increasing the rate of transitions out of the *Nanog*-low state and inhibiting the reverse transition. Third, as shown for *Nanog*, 2i increases burst frequency and reduces mixing times, effectively speeding up the intrastate equilibration rate within the cell population.

DNA Methylation Modulates Metastability

Previous work has shown that in addition to reducing heterogeneity, 2i also diminishes global levels of DNA methylation (Ficz et al., 2011; Habibi et al., 2013; Leitch et al., 2013). While the *Rex1*-high and -low states appear differentially methylated (Figures 3B–3E), it remains unclear whether methylation plays a functional role in stabilizing these states. To address this issue, we used a triple-knockout (TKO) cell line lacking the active DNA methyltransferases Dnmt1, Dnmt3A, and Dnmt3B (Tsumura et al., 2006). We compared the expression distribution of *Rex1*, *Nanog*, and *Esrrb* in TKO cells to its parental line using smFISH. The TKO cell lines had 35% ± 2% fewer cells in the *Rex1*-low state (Figure 5E), with similar results observed for *Nanog* and *Esrrb*. This change did not reflect global upregulation of all genes, as expression of the houskeeping gene *SDHA* was indistinguishable between the two cell lines. These results suggest that DNA methyltransferases increase the population of the *Rex1*-low state.

To see if these results could be recapitulated with acute rather than chronic perturbations to methylation, we assayed changes in heterogeneity in *Rex1*-dGFP reporter cells exposed to 70 nM 5-azacytidine (5-aza), an inhibitor of DNA methylation. Within 6 days, the number of cells in the *Rex1*-low state diminished by more than half from 29% to 13% of all cells (Figure 5F). Thus, acute as well as chronic methylation inhibition reduced the occupancy of the low state.

Finally, we asked whether methylation was similarly required for cells to return to the low state after removal of 2i from 2i + serum + LIF conditions. The *Rex1*-low population began to emerge within 48 hr of 2i removal (Figure 5G). However, when 2i was removed and replaced with 5-aza, the emergence of *Rex1*-low cells was severely delayed and diminished. After 6 days, 5-aza-treated cells only showed 6% *Rex1*-low cells, compared to 25% in DMSO-treated cells. Together, these results suggest that methylation is required for normal exit from the 2i state. Reduced methylation in 2i thus contributes to the stability of the 2i "ground state" (Ficz et al., 2011; Habibi et al., 2013; Leitch et al., 2013; Marks et al., 2012).

DISCUSSION

Recent work on ESC biology from a systems perspective has highlighted the apparent complexity and strong interconnectivity of the circuit governing pluripotency (Chen et al., 2008; Wang et al., 2006). But it has been unclear how variably this circuit behaves in different cells and to what extent population average measurement techniques may obscure its single-cell dynamics. Because gene expression is a stochastic process, levels of both mRNA and protein in each cell are effectively random variables, best characterized by their distributions. The framework of stochastic gene expression provides a tool to more rigorously and quantitatively separate stochastic fluctuations inherent to gene expression from variation due to multiple cell states specified by the underlying transcriptional and signaling circuit. While the simplified model of bursty transcription used here can explain the data, other models, including the "telegraph" model of transcription, may provide other insights (Iyer-Biswas et al., 2009; Peccoud and Ycart, 1995).

Our data suggest that heterogeneity emerges in three distinct ways: First, gene expression is inherently noisy, occurring in stochastic bursts, even in genes such as Oct4 that are distributed in a relatively uniform fashion. Second, cells switch stochastically between distinct states that impact the expression of many genes in a coordinated manner. Third, "long-tailed" regulators such as *Prdm14*, *Tbx3*, and *Dppa3* are uncorrelated with one another and are distributed in a manner consistent with low burst frequencies and large burst sizes, leading to very high variability. Live-cell imaging will be required to determine the absolute burst kinetics for these genes. However, an mRNA distribution in which only a small subpopulation of cells exhibit a large number

(D) Mixing time in each condition is estimated from autocorrelation, A(τ), of production rate ranks shown in Figure S5D, right panels. Red, *Nanog*-high in serum + LIF; purple, *Nanog*-SH in 2i + serum + LIF. Error bars: standard deviation, bootstrap method.

(E) Comparison of transcriptional heterogeneity between Dnmt TKO (black line) and the parental line (blue bars) as measured by smFISH for *Rex1*, *Nanog*, *Esrrb*, *and SDHA*. Note that for *Rex1*/*Nanog*/*Essrb*, there are fewer "off" cells in the leftmost bins for the TKO than WT.

(F) *Rex1*-dGFP distribution as measured by flow cytometry grown in serum + LIF with 5-aza or DMSO (carrier control). Time points were taken after 2, 4, and 6 days.

(G) Cells were grown in 2i + serum + LIF and subsequently replated into serum + LIF with 5-aza or DMSO (carrier control). Time points were taken after 2, 4, and 6 days. GFP levels were measured by flow cytometry.

of mRNA molecules for a particular gene need not, and most likely does not, indicate a distinct cellular state. Moreover, infrequent bursting may provide a potential mechanism for stochastic priming of cell fate decision-making (Maamar et al., 2007; Süel et al., 2006). Further investigation of this possibility will require determining whether these bursts propagate to influence subsequent cell fate decision-making events (Dunlop et al., 2008; Pedraza and van Oudenaarden, 2005).

The data above implicate methylation as a key regulatory mechanism affecting stochastic switching between states. Methylation was previously explored in ESCs at the population level (Ficz et al., 2011; Fouse et al., 2008; Habibi et al., 2013; Ito et al., 2010; Leitch et al., 2013; Mohn et al., 2008), but it remained unclear whether methylation itself contributes to heterogeneity (Chambers et al., 2007; Hayashi et al., 2008; Toyooka et al., 2008; Yamaji et al., 2013). smFISH data revealed a strong reciprocal relationship between the hydroxymethylase *Tet1* and the DNA methyltransferase *Dnmt3b*, with *Tet1* expressed more highly in the *Rex1*-high state and *Dnmt3b* expressed more highly in the *Rex1*-low state. This difference in expression correlates with a differential global DNA methylation and in the methylation of the promoters of key pluripotency regulators. Finally, methylation appears to be functionally required for transitions, since either genetic deletion of DNA methyltransferases or pharmacological inhibition both impact the populations of the two cell states and the underlying dynamics of state-switching (Figures 5E–5G). It will be interesting to see whether methylation plays similar functional roles in other stochastic state-switching systems.

These data provoke further questions about the molecular mechanisms through which methylation is regulated and how it modulates metastability. For example, while known methyl binding proteins that aid in methylation-dependent chromatin compaction and silencing are expressed in ESCs (Marks et al., 2012), DNA methylation may also inhibit binding of transcription factors (Shukla et al., 2011; Takizawa et al., 2001; You et al., 2011) and can control mRNA isoform selection via alternative splicing (Shukla et al., 2011). The *Esrrb* gene, whose activity is central to maintenance of pluripotency (Festuccia et al., 2012; Martello et al., 2012), may provide a good model system to investigate the effects of methylation, since its methylation levels and expression levels are both strongly state dependent. Regulation of this methylation likely involves Prdm14, which is known to inhibit Dnmt3b expression (Ficz et al., 2013; Grabole et al., 2013; Habibi et al., 2013; Leitch et al., 2013; Ma et al., 2011; Yamaji et al., 2013). Given the long-tailed expression pattern of *Prdm14* observed here in serum + LIF and its strong upregulation in 2i, it will be interesting to see how much of the variation in *Dnmt3b*/*Tet1*, and methylation more generally, can be attributed to bursty expression of *Prdm14*.

Previous studies of ESC gene expression dynamics have focused on the equilibration of FACS-sorted subpopulations of high and low *Nanog* and *Rex1* expression (Chambers et al., 2007; Toyooka et al., 2008). Two groups explored transcriptional circuit models to explain the long timescales of state-switching dynamics (Glauche et al., 2010; Kalmar et al., 2009). These included noise-induced bistable switches, oscillators, and noise-excitable circuits (Furusawa and Kaneko, 2012). Our dynamic data demonstrate that both *Nanog*-high and *Nanog*-low states in serum + LIF conditions typically persist for $\gtrsim 4$ cell cycles, and that state-switching events are abrupt at the level of promoter activity. Depending on protein and mRNA half-lives, the timescale of resulting protein level changes may follow somewhat more slowly. State-switching events are also infrequent (<0.1 per cell cycle) and uncorrelated between sister cells. Together, these findings appear incompatible with oscillatory or excitable models, which predict deterministic state-switching or an unstable excited state, respectively, but are consistent with the stochastic bistable switch model previously proposed (Kalmar et al., 2009). These properties could make this state-switching system a useful model for understanding the circuit level dynamics of spontaneous cell state transitions in single cells.

Several competing explanations were proposed for the apparent heterogeneity in *Nanog* expression in serum + LIF conditions. These models suggest heterogeneity is an artifact of knockin reporters (Faddah et al., 2013), or that it arises at least in part from monoallelic regulation (Miyanari and Torres-Padilla, 2012) or is manifested biallelically (Filipczyk et al., 2013; Hansen and van Oudenaarden, 2013). Our smFISH data support the existence of *Nanog* expression heterogeneity in wild-type cells in a standard feeder-free culture condition. Further, both static and dynamic measurements indicate that intrastate heterogeneity in *Nanog* is consistent with bursty transcription, with a relatively low burst frequency of $\sim$0.39 burst/hr. Thus, active transcriptional loci analyzed by smFISH against nascent transcripts (Miyanari and Torres-Padilla, 2012) would be expected to "flicker" on and off stochastically due to bursting. Such bursting could also cause the misleading appearance of weak correlations between alleles in static snapshots and in measurements based on destabilized fluorescent reporters. On the other hand, the *Nanog* protein fusion reporters analyzed by Filipczyk et al. showed correlated static levels between alleles, likely because the longer lifetime of their reporters allowed integration of signal over many transcriptional bursts, and because transitions between cellular states are rare and affect both alleles in a correlated fashion. The results of Faddah et al. with dual transcriptional reporters similarly showed general correlations between the two alleles, consistent with the smFISH correlations shown here (Figures S1E and S4B). Taken together, these previous studies and data presented here appear to converge on a relatively simple picture of heterogeneity based on two states and stochastic bursting.

The quantitative measurement and analysis platform described above should enable further investigation of the structure of static and dynamic heterogeneity in single ESCs. With the advent of higher-dimensionality smFISH (Lubeck et al., 2014; Lubeck and Cai, 2012), single-cell RNA-seq, and microfluidic high-throughput qPCR approaches, as well as improved methods for rapidly and accurately constructing knockin reporters (Wang et al., 2013), it will soon be possible to explore the dynamics of ESC components in higher dimensions in individual cells, both within metastable states and during cell state transitions (Kueh et al., 2013). Ultimately, this should provide a better understanding of the dynamic architecture of cell fate transition circuits.

EXPERIMENTAL PROCEDURES

Culture Conditions and Cell Lines

E14 cells (E14Tg2a.4) obtained from Mutant Mouse Regional Resource Centers were used for smFISH studies. N_{KI}Cit cells, created by Kathrin Plath, were generated by targeting the endogenous *Nanog* locus in V6.5 cells with H2B-Citrine-IRES-Neo-SV40pA (Figure S4A). N_{KI}Cit+Cer cells were generated by randomly integrating into N_{KI}Cit cells a linearized PGK-H2B-Cerulean-BGHpA-SV40-Hygro-SV40pA vector. O_{BAC}Cer cells were generated by randomly integrating into E14 cells (a kind gift from Bill Skarnes and Peri Tate) a linearized bacterial artificial chromosome (BAC) containing the *Oct4* locus (BACPAC [CHORI]), in which H2B-Cerulean-SV40pA-PGK-Neo-BGHpA was inserted before the coding sequence (Figure S4A). Rex1-dGFP cells were kindly provided by the Austin Smith lab (Wray et al., 2011). The *Dnmt* TKO cell line was provided by the RIKEN BRC through the National Bio-Resource Project of the MEXT, Japan. All cells were maintained on gelatin-coated dishes without feeders.

smFISH Hybridization, Imaging, and Analysis

The RNA FISH protocol from Raj et al., 2008 was adapted for fixed cells in suspension. See Supplemental Experimental Procedures for details. Semiautomated dot detection and segmentation were performed using custom Matlab software. A Laplacian-of-Gaussian (LoG) Kernel was used to score potential dots across all cells. The distribution of these scores across all potential dots was thresholded by Otsu's method to discriminate between true dots and background dots (see Figures S1A–S1D). Please see Table S2 for the smFISH probe sequences used in this study.

mRNA Distribution Fitting

The Negative Binomial Distribution is defined as

$$\boldsymbol{P}(n,r,p_o)=\binom{n+r-1}{n}p_0^r(1-p_o)^n,$$

where n = number of transcripts per cell, p_0 = probability of transition from the "on" promoter state to the "off" promoter state, and r = number of bursting events per mRNA half-life. The average burst size is computed as $b=(1-p_0)/p_0$. Using this model, individual mRNA distributions were fit using maximum likelihood estimation. To discriminate between unimodal and bimodal fits, two tests were used to ensure that the improvement of the fit is counterbalanced by the additional degrees of freedom from the added parameters. To be considered bimodal, a distribution was required to pass both Akaike Information Criteria (AIC) and the log-likelihood ratio test ($p < 0.05$).

Fluorescence Time-Lapse Microscopy and Data analysis

Reporter cells were mixed with unlabeled parental cells at 1:9 ratio and plated at a total density of 20,000 cells/cm^2 on glass-bottom plates (MatTek) coated with human laminin-511 (BioLamina) >12 hr before imaging. Images were acquired every 15 min for ~50 hr with daily medium change. Cells were segmented and tracked from the acquired images using our own Matlab code (see Supplemental Information for image analysis methods).

2i Perturbation and Analysis

For 2i treatment we supplemented serum + LIF media with MEK inhibitor PD0325901 at 1 μM and GSK3 inhibitor CH99021 at 3 μM. Cells grown in serum + LIF media were treated with 2i for 6 days before harvesting for smFISH assay and imaging for movies.

Methylation Analysis and Perturbation

RRBS preparation and high-throughput sequencing were performed by Zymo Research. RRBS data were processed using Bismark and Galaxy. To be included in the analysis, each CpG had to have at least five reads. For perturbation experiments, 5-aza (Sigma) was used at a final concentration of 70 nM. 5mC ELISA was performed with ELISA 5mC kit (Zymo).

ACCESSION NUMBERS

Sequencing data have been deposted in NCBI's GEO under accession number GSE58396.

SUPPLEMENTAL INFORMATION

Supplemental Information includes Supplemental Experimental Procedures, five figures, two tables, and four movies and can be found with this article online at http://dx.doi.org/10.1016/j.molcel.2014.06.029.

AUTHOR CONTRIBUTIONS

Z.S.S. and J.Y. contributed equally and are listed alphabetically. Z.S.S., J.Y., J.T., L.C., M.A.S., and M.B.E. conceived experiments. Z.S.S., J.Y., J.T., and J.A.H. performed experiments and analyzed data, with Z.S.S. leading smFISH and methylation experiments and J.Y. leading the movie experiments and modeling. A.A. contributed computational algorithms. M.A.S. and M.B.E. supervised research. Z.S.S., J.T., J.Y., and M.B.E. wrote the manuscript with substantial input from all authors.

ACKNOWLEDGMENTS

We thank Jordi Garcia-Ojalvo, Xiling Shen, Georg Seelig, Arjun Raj, and David Sprinzak for helpful comments on the manuscript; the Kathrin Plath Lab, the Austin Smith Lab, and RIKEN for kindly providing reporter and knockout cell lines; and the Caltech FACS Facility for assistance with cell sorting. This work was supported by the National Institutes of Health grants R01HD075605A, R01GM086793A, and P50GM068763; the Weston Havens Foundation; Human Frontiers Science Program; the Packard Foundation; a Wellcome Trust Investigators Grant to M.A.S.; and a KAUST, APART, and CIRM Fellowship to J.T. This work is funded by the Gordon and Betty Moore Foundation through Grant GBMF2809 to the Caltech Programmable Molecular Technology Initiative.

Received: October 18, 2013
Revised: April 4, 2014
Accepted: June 18, 2014
Published: July 17, 2014

REFERENCES

Auernhammer, C.J., Bousquet, C., and Melmed, S. (1999). Autoregulation of pituitary corticotroph SOCS-3 expression: characterization of the murine SOCS-3 promoter. Proc. Natl. Acad. Sci. USA *96*, 6964–6969.

Blake, W.J., KAErn, M., Cantor, C.R., and Collins, J.J. (2003). Noise in eukaryotic gene expression. Nature *422*, 633–637.

Borgel, J., Guibert, S., Li, Y., Chiba, H., Schübeler, D., Sasaki, H., Forné, T., and Weber, M. (2010). Targets and dynamics of promoter DNA methylation during early mouse development. Nat. Genet. *42*, 1093–1100.

Brewster, R.C., Weinert, F.M., Garcia, H.G., Song, D., Rydenfelt, M., and Phillips, R. (2014). The transcription factor titration effect dictates level of gene expression. Cell *156*, 1312–1323.

Cai, L., Friedman, N., and Xie, X.S. (2006). Stochastic protein expression in individual cells at the single molecule level. Nature *440*, 358–362.

Canham, M.A., Sharov, A.A., Ko, M.S.H., and Brickman, J.M. (2010). Functional heterogeneity of embryonic stem cells revealed through translational amplification of an early endodermal transcript. PLoS Biol. *8*, e1000379.

Chambers, I., Silva, J., Colby, D., Nichols, J., Nijmeijer, B., Robertson, M., Vrana, J., Jones, K., Grotewold, L., and Smith, A. (2007). Nanog safeguards pluripotency and mediates germline development. Nature *450*, 1230–1234.

Chang, H.H., Hemberg, M., Barahona, M., Ingber, D.E., and Huang, S. (2008). Transcriptome-wide noise controls lineage choice in mammalian progenitor cells. Nature *453*, 544–547.

Chen, X., Xu, H., Yuan, P., Fang, F., Huss, M., Vega, V.B., Wong, E., Orlov, Y.L., Zhang, W., Jiang, J., et al. (2008). Integration of external signaling pathways with the core transcriptional network in embryonic stem cells. Cell *133*, 1106–1117.

Choi, P.J., Cai, L., Frieda, K., and Xie, X.S. (2008). A stochastic single-molecule event triggers phenotype switching of a bacterial cell. Science *322*, 442–446.

Dunlop, M.J., Cox, R.S., 3rd, Levine, J.H., Murray, R.M., and Elowitz, M.B. (2008). Regulatory activity revealed by dynamic correlations in gene expression noise. Nat. Genet. *40*, 1493–1498.

Eldar, A., and Elowitz, M.B. (2010). Functional roles for noise in genetic circuits. Nature *467*, 167–173.

Elowitz, M.B., Levine, A.J., Siggia, E.D., and Swain, P.S. (2002). Stochastic gene expression in a single cell. Science *297*, 1183–1186.

Faddah, D.A., Wang, H., Cheng, A.W., Katz, Y., Buganim, Y., and Jaenisch, R. (2013). Single-cell analysis reveals that expression of nanog is biallelic and equally variable as that of other pluripotency factors in mouse ESCs. Cell Stem Cell *13*, 23–29.

Festuccia, N., Osorno, R., Halbritter, F., Karwacki-Neisius, V., Navarro, P., Colby, D., Wong, F., Yates, A., Tomlinson, S.R., and Chambers, I. (2012). Esrrb is a direct Nanog target gene that can substitute for Nanog function in pluripotent cells. Cell Stem Cell *11*, 477–490.

Ficz, G., Branco, M.R., Seisenberger, S., Santos, F., Krueger, F., Hore, T.A., Marques, C.J., Andrews, S., and Reik, W. (2011). Dynamic regulation of 5-hydroxymethylcytosine in mouse ES cells and during differentiation. Nature *473*, 398–402.

Ficz, G., Hore, T.A., Santos, F., Lee, H.J., Dean, W., Arand, J., Krueger, F., Oxley, D., Paul, Y.-L., Walter, J., et al. (2013). FGF signaling inhibition in ESCs drives rapid genome-wide demethylation to the epigenetic ground state of pluripotency. Cell Stem Cell *13*, 351–359.

Filipczyk, A., Gkatzis, K., Fu, J., Hoppe, P.S., Lickert, H., Anastassiadis, K., and Schroeder, T. (2013). Biallelic expression of nanog protein in mouse embryonic stem cells. Cell Stem Cell *13*, 12–13.

Fouse, S.D., Shen, Y., Pellegrini, M., Cole, S., Meissner, A., Van Neste, L., Jaenisch, R., and Fan, G. (2008). Promoter CpG methylation contributes to ES cell gene regulation in parallel with Oct4/Nanog, PcG complex, and histone H3 K4/K27 trimethylation. Cell Stem Cell *2*, 160–169.

Friedman, N., Cai, L., and Xie, X.S. (2006). Linking stochastic dynamics to population distribution: an analytical framework of gene expression. Phys. Rev. Lett. *97*, 168302.

Furusawa, C., and Kaneko, K. (2012). A dynamical-systems view of stem cell biology. Science *338*, 215–217.

Glauche, I., Herberg, M., and Roeder, I. (2010). Nanog variability and pluripotency regulation of embryonic stem cells—insights from a mathematical model analysis. PLoS ONE *5*, e11238.

Grabole, N., Tischler, J., Hackett, J.A., Kim, S., Tang, F., Leitch, H.G., Magnúsdóttir, E., and Surani, M.A. (2013). Prdm14 promotes germline fate and naive pluripotency by repressing FGF signalling and DNA methylation. EMBO Rep. *14*, 629–637.

Guo, G., Huss, M., Tong, G.Q., Wang, C., Li Sun, L., Clarke, N.D., and Robson, P. (2010). Resolution of cell fate decisions revealed by single-cell gene expression analysis from zygote to blastocyst. Dev. Cell *18*, 675–685.

Gupta, P.B., Fillmore, C.M., Jiang, G., Shapira, S.D., Tao, K., Kuperwasser, C., and Lander, E.S. (2011). Stochastic state transitions give rise to phenotypic equilibrium in populations of cancer cells. Cell *146*, 633–644.

Habibi, E., Brinkman, A.B., Arand, J., Kroeze, L.I., Kerstens, H.H.D., Matarese, F., Lepikhov, K., Gut, M., Brun-Heath, I., Hubner, N.C., et al. (2013). Whole-genome bisulfite sequencing of two distinct interconvertible DNA methylomes of mouse embryonic stem cells. Cell Stem Cell *13*, 360–369.

Hackett, J.A., Sengupta, R., Zylicz, J.J., Murakami, K., Lee, C., Down, T.A., and Surani, M.A. (2013). Germline DNA demethylation dynamics and imprint erasure through 5-hydroxymethylcytosine. Science *339*, 448–452.

Hansen, C.H., and van Oudenaarden, A. (2013). Allele-specific detection of single mRNA molecules in situ. Nat. Methods *10*, 869–871.

Hayashi, K., Lopes, S.M.C. de S., Tang, F., and Surani, M.A. (2008). Dynamic equilibrium and heterogeneity of mouse pluripotent stem cells with distinct functional and epigenetic states. Cell Stem Cell *3*, 391–401.

Ito, S., D'Alessio, A.C., Taranova, O.V., Hong, K., Sowers, L.C., and Zhang, Y. (2010). Role of Tet proteins in 5mC to 5hmC conversion, ES-cell self-renewal and inner cell mass specification. Nature *466*, 1129–1133.

Iyer-Biswas, S., Hayot, F., and Jayaprakash, C. (2009). Stochasticity of gene products from transcriptional pulsing. Phys. Rev. E Stat. Nonlin. Soft Matter Phys. *79*, 031911–031911.

Jaitin, D.A., Kenigsberg, E., Keren-Shaul, H., Elefant, N., Paul, F., Zaretsky, I., Mildner, A., Cohen, N., Jung, S., Tanay, A., and Amit, I. (2014). Massively parallel single-cell RNA-seq for marker-free decomposition of tissues into cell types. Science *343*, 776–779.

Kalmar, T., Lim, C., Hayward, P., Muñoz-Descalzo, S., Nichols, J., Garcia-Ojalvo, J., and Martinez Arias, A. (2009). Regulated fluctuations in nanog expression mediate cell fate decisions in embryonic stem cells. PLoS Biol. *7*, e1000149.

Koh, K.P., Yabuuchi, A., Rao, S., Huang, Y., Cunniff, K., Nardone, J., Laiho, A., Tahiliani, M., Sommer, C.A., Mostoslavsky, G., et al. (2011). Tet1 and Tet2 regulate 5-hydroxymethylcytosine production and cell lineage specification in mouse embryonic stem cells. Cell Stem Cell *8*, 200–213.

Kueh, H.Y., Champhekar, A., Nutt, S.L., Elowitz, M.B., and Rothenberg, E.V. (2013). Positive feedback between PU.1 and the cell cycle controls myeloid differentiation. Science *341*, 670–673.

Lander, A.D., Gokoffski, K.K., Wan, F.Y.M., Nie, Q., and Calof, A.L. (2009). Cell lineages and the logic of proliferative control. PLoS Biol. *7*, e15.

Leitch, H.G., McEwen, K.R., Turp, A., Encheva, V., Carroll, T., Grabole, N., Mansfield, W., Nashun, B., Knezovich, J.G., Smith, A., et al. (2013). Naive pluripotency is associated with global DNA hypomethylation. Nat. Struct. Mol. Biol. *20*, 311–316.

Locke, J.C.W., and Elowitz, M.B. (2009). Using movies to analyse gene circuit dynamics in single cells. Nat. Rev. Microbiol. *7*, 383–392.

Lubeck, E., and Cai, L. (2012). Single-cell systems biology by super-resolution imaging and combinatorial labeling. Nat. Methods *9*, 743–748.

Lubeck, E., Coskun, A.F., Zhiyentayev, T., Ahmad, M., and Cai, L. (2014). Single-cell in situ RNA profiling by sequential hybridization. Nat. Methods *11*, 360–361.

Ma, Z., Swigut, T., Valouev, A., Rada-Iglesias, A., and Wysocka, J. (2011). Sequence-specific regulator Prdm14 safeguards mouse ESCs from entering extraembryonic endoderm fates. Nat. Struct. Mol. Biol. *18*, 120–127.

Maamar, H., Raj, A., and Dubnau, D. (2007). Noise in gene expression determines cell fate in Bacillus subtilis. Science *317*, 526–529.

Macfarlan, T.S., Gifford, W.D., Driscoll, S., Lettieri, K., Rowe, H.M., Bonanomi, D., Firth, A., Singer, O., Trono, D., and Pfaff, S.L. (2012). Embryonic stem cell potency fluctuates with endogenous retrovirus activity. Nature *487*, 57–63.

Marks, H., Kalkan, T., Menafra, R., Denissov, S., Jones, K., Hofemeister, H., Nichols, J., Kranz, A., Stewart, A.F., Smith, A., and Stunnenberg, H.G. (2012). The transcriptional and epigenomic foundations of ground state pluripotency. Cell *149*, 590–604.

Martello, G., Sugimoto, T., Diamanti, E., Joshi, A., Hannah, R., Ohtsuka, S., Göttgens, B., Niwa, H., and Smith, A. (2012). Esrrb is a pivotal target of the Gsk3/Tcf3 axis regulating embryonic stem cell self-renewal. Cell Stem Cell *11*, 491–504.

Meissner, A., Mikkelsen, T.S., Gu, H., Wernig, M., Hanna, J., Sivachenko, A., Zhang, X., Bernstein, B.E., Nusbaum, C., Jaffe, D.B., et al. (2008). Genome-scale DNA methylation maps of pluripotent and differentiated cells. Nature *454*, 766–770.

Miyanari, Y., and Torres-Padilla, M.-E. (2012). Control of ground-state pluripotency by allelic regulation of Nanog. Nature *483*, 470–473.

Mohn, F., Weber, M., Rebhan, M., Roloff, T.C., Richter, J., Stadler, M.B., Bibel, M., and Schübeler, D. (2008). Lineage-specific polycomb targets and de novo

DNA methylation define restriction and potential of neuronal progenitors. Mol. Cell *30*, 755–766.

Okano, M., Bell, D.W., Haber, D.A., and Li, E. (1999). DNA methyltransferases Dnmt3a and Dnmt3b are essential for de novo methylation and mammalian development. Cell *99*, 247–257.

Ozbudak, E.M., Thattai, M., Kurtser, I., Grossman, A.D., and van Oudenaarden, A. (2002). Regulation of noise in the expression of a single gene. Nat. Genet. *31*, 69–73.

Paulsson, J., and Ehrenberg, M. (2000). Random signal fluctuations can reduce random fluctuations in regulated components of chemical regulatory networks. Phys. Rev. Lett. *84*, 5447–5450.

Paulsson, J., Berg, O.G., and Ehrenberg, M. (2000). Stochastic focusing: fluctuation-enhanced sensitivity of intracellular regulation. Proc. Natl. Acad. Sci. USA *97*, 7148–7153.

Peccoud, J., and Ycart, B. (1995). Markovian modeling of gene-product synthesis. Theor. Popul. Biol. *48*, 222–234.

Pedraza, J.M., and van Oudenaarden, A. (2005). Noise propagation in gene networks. Science *307*, 1965–1969.

Raj, A., Peskin, C.S., Tranchina, D., Vargas, D.Y., and Tyagi, S. (2006). Stochastic mRNA synthesis in mammalian cells. PLoS Biol. *4*, e309.

Raj, A., van den Bogaard, P., Rifkin, S.A., van Oudenaarden, A., and Tyagi, S. (2008). Imaging individual mRNA molecules using multiple singly labeled probes. Nat. Methods *5*, 877–879.

Reik, W. (2007). Stability and flexibility of epigenetic gene regulation in mammalian development. Nature *447*, 425–432.

Rompolas, P., Mesa, K.R., and Greco, V. (2013). Spatial organization within a niche as a determinant of stem-cell fate. Nature *502*, 513–518.

Rosenfeld, N., Young, J.W., Alon, U., Swain, P.S., and Elowitz, M.B. (2005). Gene regulation at the single-cell level. Science *307*, 1962–1965.

Schübeler, D., Lorincz, M.C., Cimbora, D.M., Telling, A., Feng, Y.Q., Bouhassira, E.E., and Groudine, M. (2000). Genomic targeting of methylated DNA: influence of methylation on transcription, replication, chromatin structure, and histone acetylation. Mol. Cell. Biol. *20*, 9103–9112.

Shahrezaei, V., and Swain, P.S. (2008). Analytical distributions for stochastic gene expression. Proc. Natl. Acad. Sci. USA *105*, 17256–17261.

Shalek, A.K., Satija, R., Adiconis, X., Gertner, R.S., Gaublomme, J.T., Raychowdhury, R., Schwartz, S., Yosef, N., Malboeuf, C., Lu, D., et al. (2013). Single-cell transcriptomics reveals bimodality in expression and splicing in immune cells. Nature *498*, 236–240.

Sharova, L.V., Sharov, A.A., Nedorezov, T., Piao, Y., Shaik, N., and Ko, M.S. (2009). Database for mRNA half-life of 19 977 genes obtained by DNA microarray analysis of pluripotent and differentiating mouse embryonic stem cells. DNA Res. *16*, 45–58.

Shukla, S., Kavak, E., Gregory, M., Imashimizu, M., Shutinoski, B., Kashlev, M., Oberdoerffer, P., Sandberg, R., and Oberdoerffer, S. (2011). CTCF-promoted RNA polymerase II pausing links DNA methylation to splicing. Nature *479*, 74–79.

Sigal, A., Milo, R., Cohen, A., Geva-Zatorsky, N., Klein, Y., Liron, Y., Rosenfeld, N., Danon, T., Perzov, N., and Alon, U. (2006). Variability and memory of protein levels in human cells. Nature *444*, 643–646.

Singh, A.M., Hamazaki, T., Hankowski, K.E., and Terada, N. (2007). A heterogeneous expression pattern for Nanog in embryonic stem cells. Stem Cells *25*, 2534–2542.

Smith, Z.D., Chan, M.M., Mikkelsen, T.S., Gu, H., Gnirke, A., Regev, A., and Meissner, A. (2012). A unique regulatory phase of DNA methylation in the early mammalian embryo. Nature *484*, 339–344.

Süel, G.M., Garcia-Ojalvo, J., Liberman, L.M., and Elowitz, M.B. (2006). An excitable gene regulatory circuit induces transient cellular differentiation. Nature *440*, 545–550.

Suter, D.M., Molina, N., Gatfield, D., Schneider, K., Schibler, U., and Naef, F. (2011). Mammalian genes are transcribed with widely different bursting kinetics. Science *332*, 472–474.

Takizawa, T., Nakashima, K., Namihira, M., Ochiai, W., Uemura, A., Yanagisawa, M., Fujita, N., Nakao, M., and Taga, T. (2001). DNA methylation is a critical cell-intrinsic determinant of astrocyte differentiation in the fetal brain. Dev. Cell *1*, 749–758.

Toyooka, Y., Shimosato, D., Murakami, K., Takahashi, K., and Niwa, H. (2008). Identification and characterization of subpopulations in undifferentiated ES cell culture. Development *135*, 909–918.

Tsumura, A., Hayakawa, T., Kumaki, Y., Takebayashi, S.-I., Sakaue, M., Matsuoka, C., Shimotohno, K., Ishikawa, F., Li, E., Ueda, H.R., et al. (2006). Maintenance of self-renewal ability of mouse embryonic stem cells in the absence of DNA methyltransferases Dnmt1, Dnmt3a and Dnmt3b. Genes Cells *11*, 805–814.

Wang, J., Rao, S., Chu, J., Shen, X., Levasseur, D.N., Theunissen, T.W., and Orkin, S.H. (2006). A protein interaction network for pluripotency of embryonic stem cells. Nature *444*, 364–368.

Wang, H., Yang, H., Shivalila, C.S., Dawlaty, M.M., Cheng, A.W., Zhang, F., and Jaenisch, R. (2013). One-step generation of mice carrying mutations in multiple genes by CRISPR/Cas-mediated genome engineering. Cell *153*, 910–918.

Wray, J., Kalkan, T., and Smith, A.G. (2010). The ground state of pluripotency. Biochem. Soc. Trans. *38*, 1027–1032.

Wray, J., Kalkan, T., Gomez-Lopez, S., Eckardt, D., Cook, A., Kemler, R., and Smith, A. (2011). Inhibition of glycogen synthase kinase-3 alleviates Tcf3 repression of the pluripotency network and increases embryonic stem cell resistance to differentiation. Nat. Cell Biol. *13*, 838–845.

Wu, H., D'Alessio, A.C., Ito, S., Xia, K., Wang, Z., Cui, K., Zhao, K., Sun, Y.E., and Zhang, Y. (2011). Dual functions of Tet1 in transcriptional regulation in mouse embryonic stem cells. Nature *473*, 389–393.

Yamaji, M., Ueda, J., Hayashi, K., Ohta, H., Yabuta, Y., Kurimoto, K., Nakato, R., Yamada, Y., Shirahige, K., and Saitou, M. (2013). PRDM14 ensures naive pluripotency through dual regulation of signaling and epigenetic pathways in mouse embryonic stem cells. Cell Stem Cell *12*, 368–382.

Yamanaka, Y., Lanner, F., and Rossant, J. (2010). FGF signal-dependent segregation of primitive endoderm and epiblast in the mouse blastocyst. Development *137*, 715–724.

You, J.S., Kelly, T.K., De Carvalho, D.D., Taberlay, P.C., Liang, G., and Jones, P.A. (2011). OCT4 establishes and maintains nucleosome-depleted regions that provide additional layers of epigenetic regulation of its target genes. Proc. Natl. Acad. Sci. USA *108*, 14497–14502.

Zenklusen, D., Larson, D.R., and Singer, R.H. (2008). Single-RNA counting reveals alternative modes of gene expression in yeast. Nat. Struct. Mol. Biol. *15*, 1263–1271.

Zong, C., So, L.-H., Sepúlveda, L.A., Skinner, S.O., and Golding, I. (2010). Lysogen stability is determined by the frequency of activity bursts from the fate-determining gene. Mol. Syst. Biol. *6*, 440.

Molecular Cell, Volume *55*

Supplemental Information

Dynamic Heterogeneity and DNA Methylation in Embryonic Stem Cells

Zakary S. Singer, John Yong, Julia Tischler, Jamie A. Hackett, Alphan Altinok, M. Azim Surani,
Long Cai, and Michael B. Elowitz

Figure S1

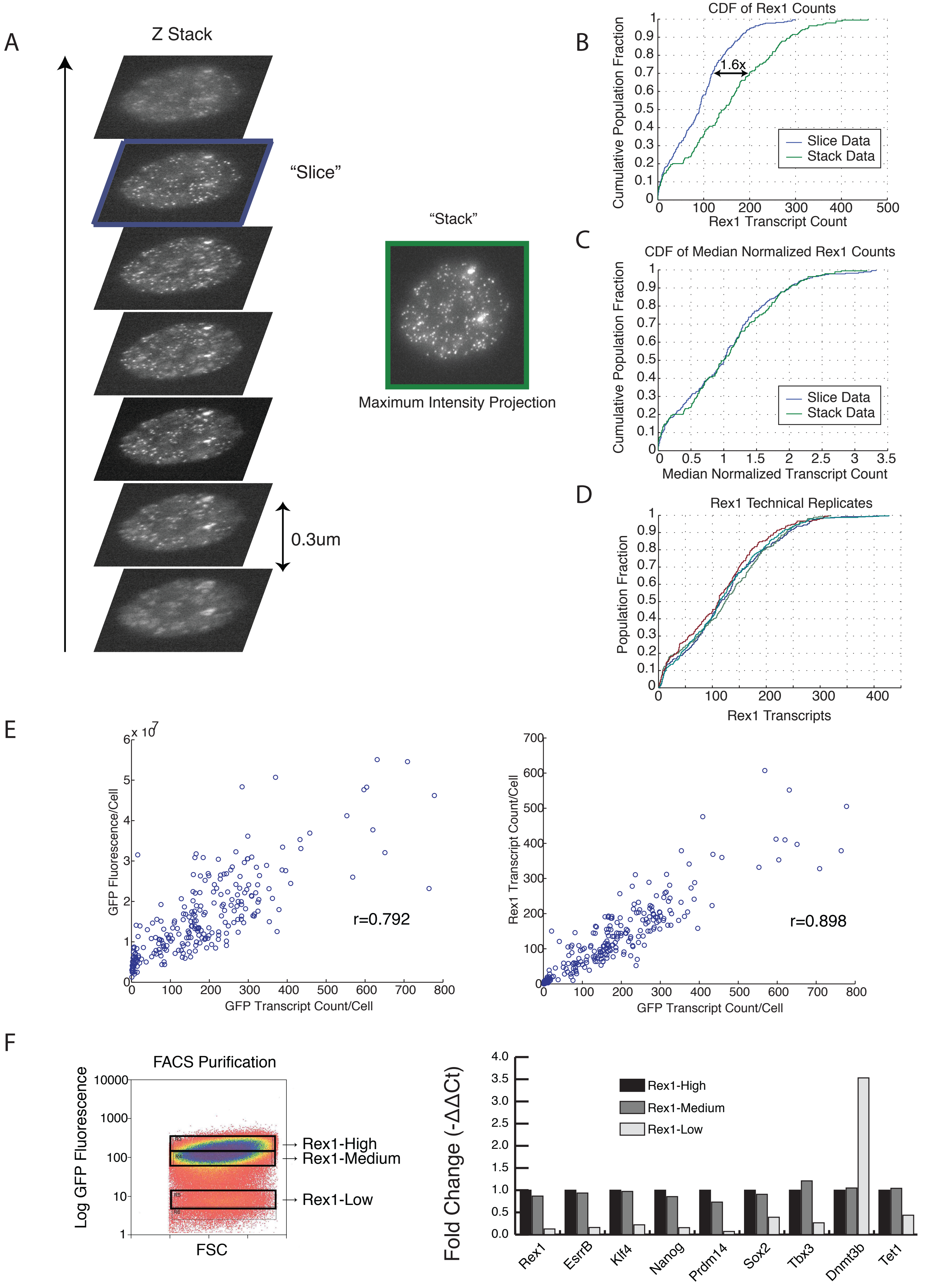
A
Z Stack
"Slice"
"Stack"
Maximum Intensity Projection
0.3um
B
CDF of Rex1 Counts
Cumulative Population Fraction
Rex1 Transcript Count
1.6x
Slice Data
Stack Data
C
CDF of Median Normalized Rex1 Counts
Cumulative Population Fraction
Median Normalized Transcript Count
Slice Data
Stack Data
D
Rex1 Technical Replicates
Population Fraction
Rex1 Transcripts
E
x 10^7
GFP Fluorescence/Cell
GFP Transcript Count/Cell
r=0.792
Rex1 Transcript Count/Cell
GFP Transcript Count/Cell
r=0.898
F
FACS Purification
Log GFP Fluorescence
FSC
Rex1-High
Rex1-Medium
Rex1-Low
Fold Change (-ΔΔCt)
Rex1-High
Rex1-Medium
Rex1-Low
Rex1
EsrrB
Klf4
Nanog
Prdm14
Sox2
Tbx3
Dnmt3b
Tet1

Figure S2.

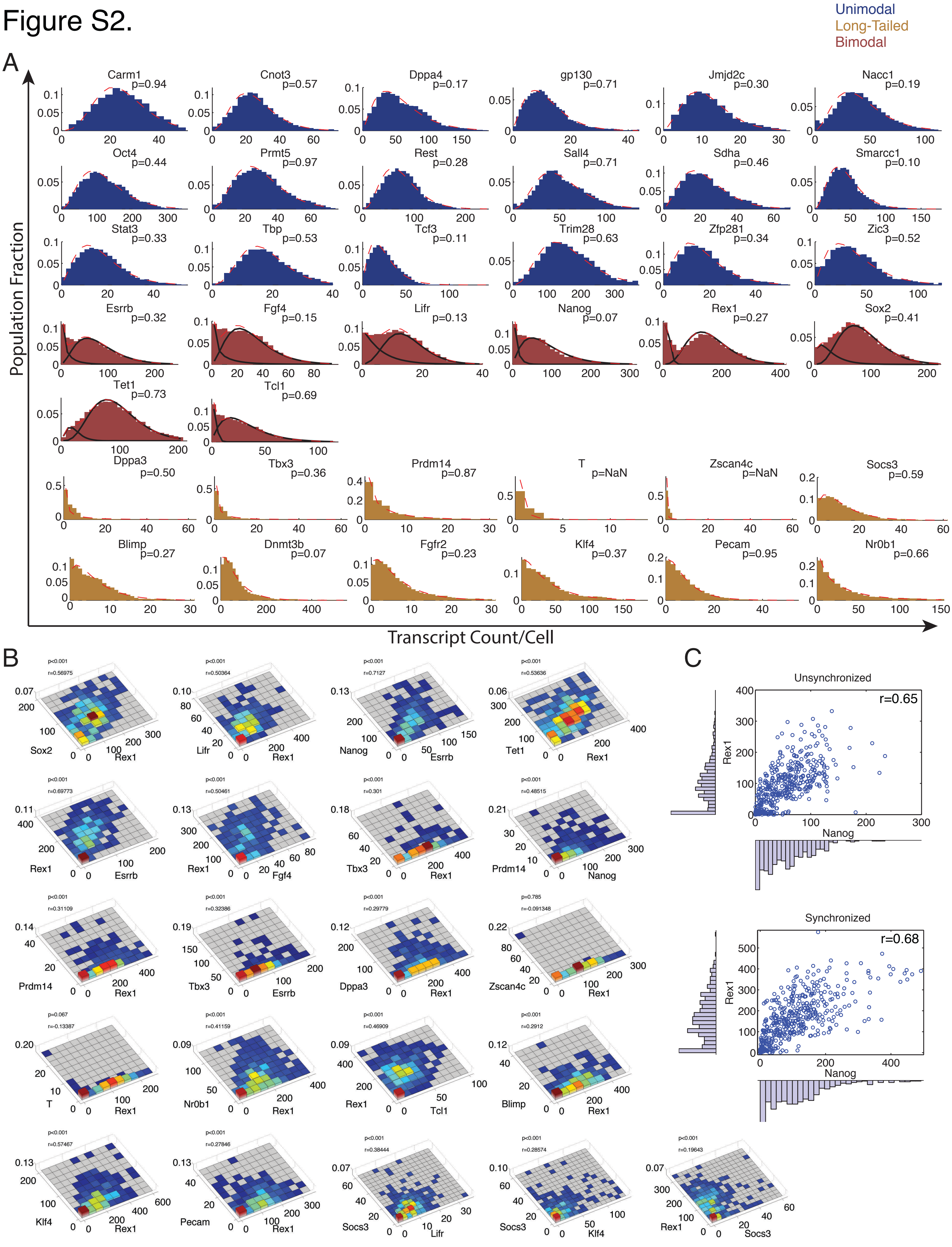

Unimodal
Long-Tailed
Bimodal
A
Population Fraction
Transcript Count/Cell
Carm1 p=0.94
Cnot3 p=0.57
Dppa4 p=0.17
gp130 p=0.71
Jmjd2c p=0.30
Nacc1 p=0.19
Oct4 p=0.44
Prmt5 p=0.97
Rest p=0.28
Sall4 p=0.71
Sdha p=0.46
Smarcc1 p=0.10
Stat3 p=0.33
Tbp p=0.53
Tcf3 p=0.11
Trim28 p=0.63
Zfp281 p=0.34
Zic3 p=0.52
Esrrb p=0.32
Fgf4 p=0.15
Lifr p=0.13
Nanog p=0.07
Rex1 p=0.27
Sox2 p=0.41
Tet1 p=0.73
Tcl1 p=0.69
Dppa3 p=0.50
Tbx3 p=0.36
Prdm14 p=0.87
T p=NaN
Zscan4c p=NaN
Socs3 p=0.59
Blimp p=0.27
Dnmt3b p=0.07
Fgfr2 p=0.23
Klf4 p=0.37
Pecam p=0.95
Nr0b1 p=0.66
B
p<0.001 r=0.56975 Sox2 Rex1
p<0.001 r=0.50364 Lifr Rex1
p<0.001 r=0.7127 Nanog Esrrb
p<0.001 r=0.53636 Tet1 Rex1
p<0.001 r=0.69773 Rex1 Esrrb
p<0.001 r=0.50461 Rex1 Fgf4
p<0.001 r=0.301 Tbx3 Rex1
p<0.001 r=0.48515 Prdm14 Nanog
p<0.001 r=0.31109 Prdm14 Rex1
p<0.001 r=0.32386 Tbx3 Esrrb
p<0.001 r=0.29779 Dppa3 Rex1
p=0.785 r=-0.091348 Zscan4c Rex1
p=0.067 r=-0.13387 T Rex1
p<0.001 r=0.41159 Nr0b1 Rex1
p<0.001 r=0.46909 Rex1 Tcl1
p<0.001 r=0.2912 Blimp Rex1
p<0.001 r=0.57467 Klf4 Rex1
p<0.001 r=0.27846 Pecam Rex1
p<0.001 r=0.38444 Socs3 Lifr
p<0.001 r=0.28574 Socs3 Klf4
p<0.001 r=0.19643 Rex1 Socs3
C
Unsynchronized
r=0.65
Rex1
Nanog
Synchronized
r=0.68
Rex1
Nanog

Figure S3

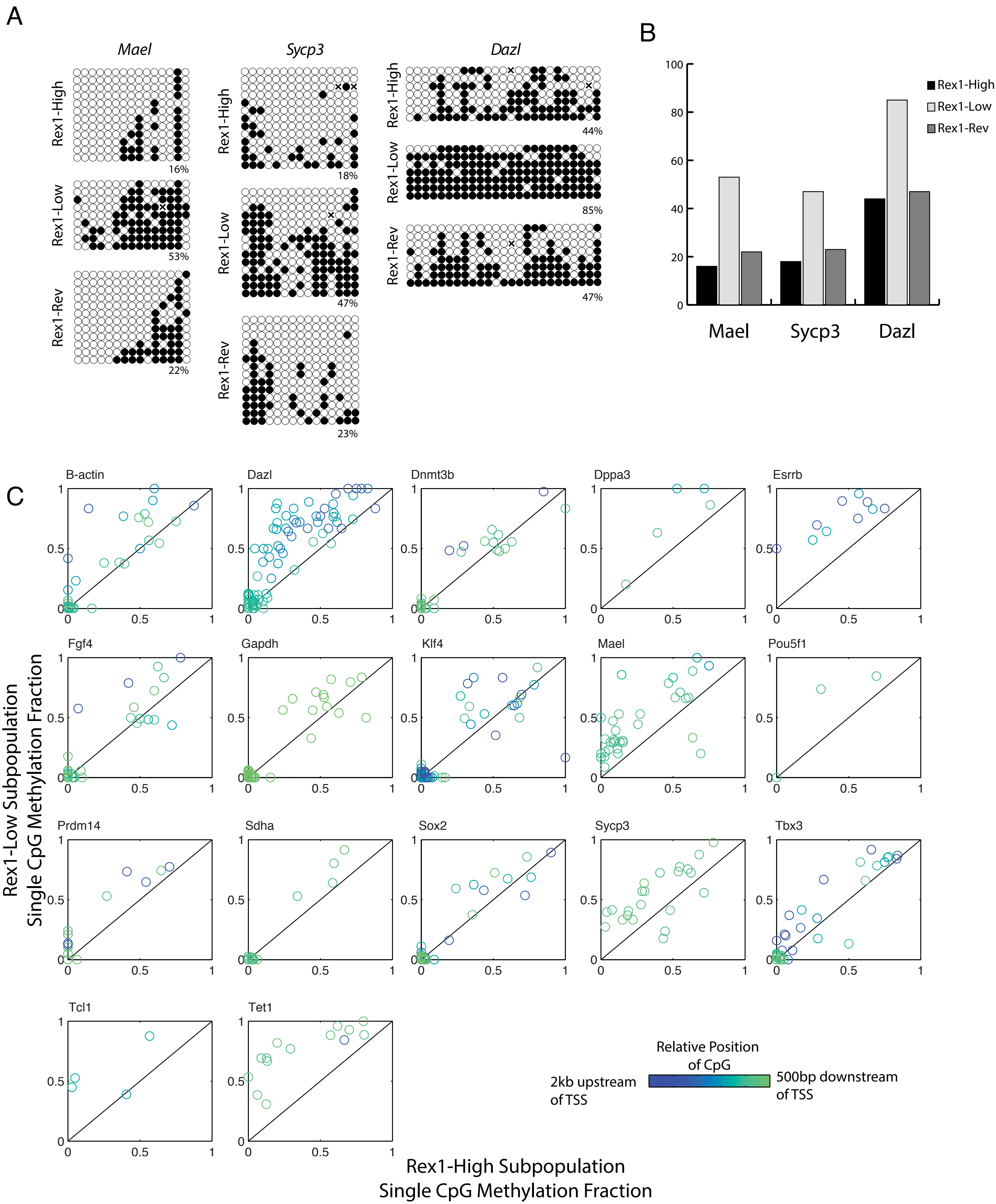

A
Mael
Sycp3
Dazl
Rex1-High
Rex1-Low
Rex1-Rev
16%
53%
22%
18%
47%
23%
44%
85%
47%
B
Rex1-High
Rex1-Low
Rex1-Rev
Mael
Sycp3
Dazl
C
B-actin
Dazl
Dnmt3b
Dppa3
Esrrb
Fgf4
Gapdh
Klf4
Mael
Pou5f1
Prdm14
Sdha
Sox2
Sycp3
Tbx3
Tcl1
Tet1
Rex1-Low Subpopulation
Single CpG Methylation Fraction
Rex1-High Subpopulation
Single CpG Methylation Fraction
Relative Position of CpG
2kb upstream of TSS
500bp downstream of TSS

Figure S4.

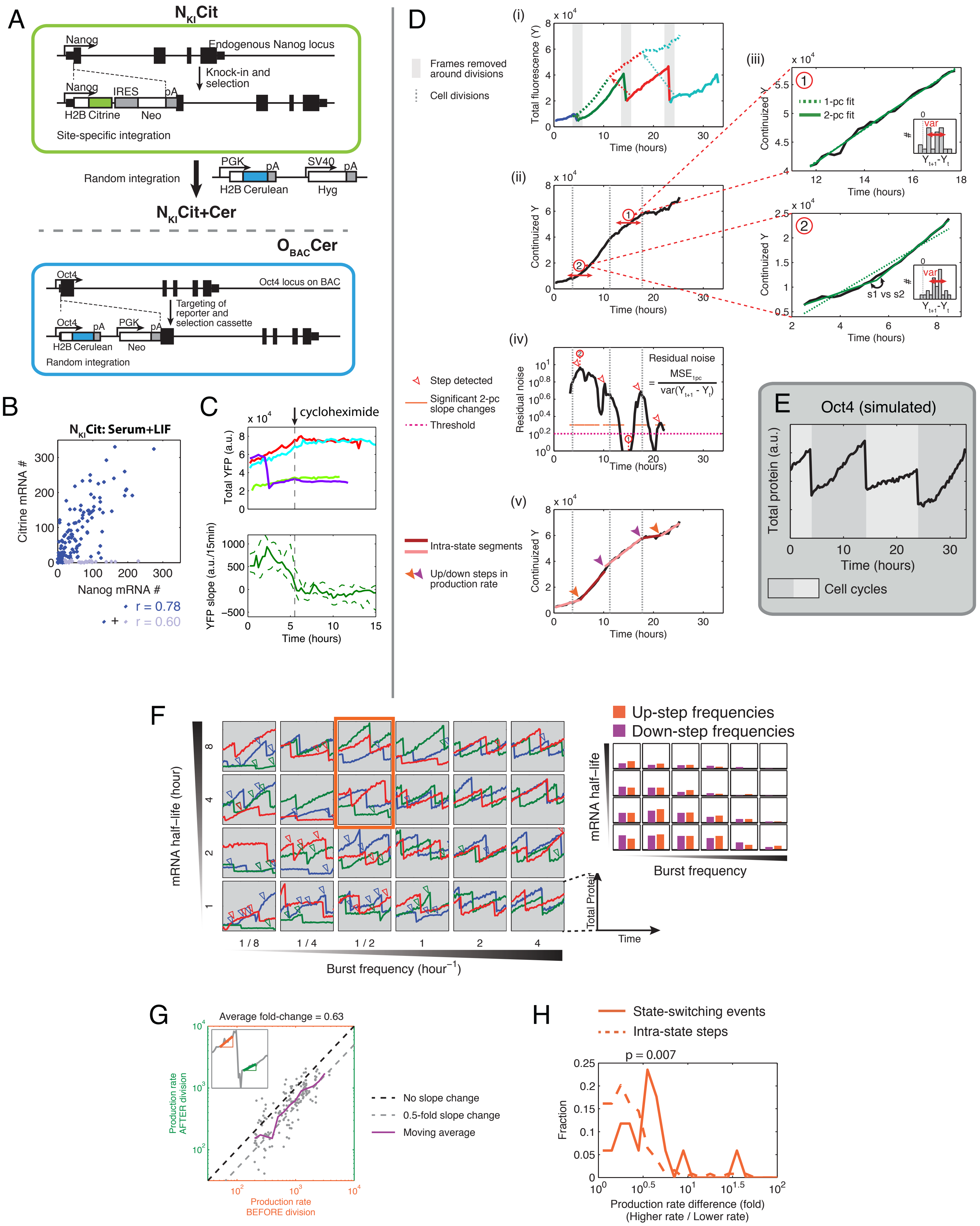

A
N_{KI}Cit
Nanog
Endogenous Nanog locus
Knock-in and selection
Nanog
IRES
pA
H2B Citrine
Neo
Site-specific integration
Random integration
PGK
pA
SV40
pA
H2B Cerulean
Hyg
N_{KI}Cit+Cer
O_{BAC}Cer
Oct4
Oct4 locus on BAC
Targeting of reporter and selection cassette
Oct4
pA
PGK
pA
H2B Cerulean
Neo
Random integration
B
N_{KI}Cit: Serum+LIF
Citrine mRNA #
Nanog mRNA #
r = 0.78
r = 0.60
C
cycloheximide
Total YFP (a.u.)
YFP slope (a.u./15min)
Time (hours)
D
Frames removed around divisions
Cell divisions
Step detected
Significant 2-pc slope changes
Threshold
Intra-state segments
Up/down steps in production rate
(i)
Total fluorescence (Y)
Time (hours)
(ii)
Continuized Y
Time (hours)
(iii)
Continuized Y
1-pc fit
2-pc fit
var
$Y_{t+1}-Y_t$
s1 vs s2
Time (hours)
(iv)
Residual noise
Residual noise
$= \frac{MSE_{1pc}}{var(Y_{t+1} - Y_t)}$
Time (hours)
(v)
Continuized Y
Time (hours)
E
Oct4 (simulated)
Total protein (a.u.)
Time (hours)
Cell cycles
F
mRNA half-life (hour)
Burst frequency (hour^{-1})
Total Protein
Time
Up-step frequencies
Down-step frequencies
mRNA half-life
Burst frequency
G
Average fold-change = 0.63
Production rate AFTER division
Production rate BEFORE division
No slope change
0.5-fold slope change
Moving average
H
State-switching events
Intra-state steps
p = 0.007
Fraction
Production rate difference (fold)
(Higher rate / Lower rate)

Figure S5.

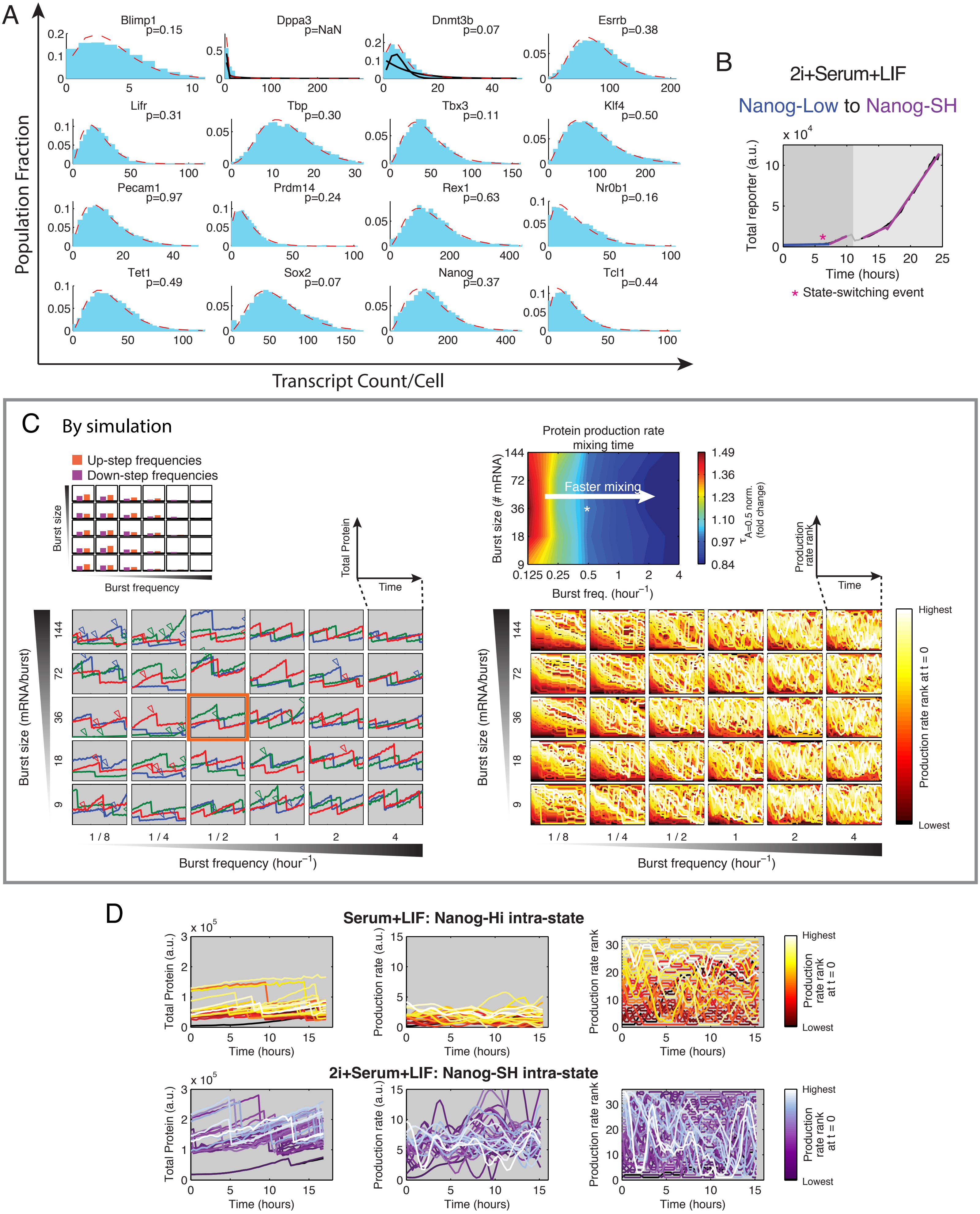
A
Population Fraction
Transcript Count/Cell
Blimp1 p=0.15
Dppa3 p=NaN
Dnmt3b p=0.07
Esrrb p=0.38
Lifr p=0.31
Tbp p=0.30
Tbx3 p=0.11
Klf4 p=0.50
Pecam1 p=0.97
Prdm14 p=0.24
Rex1 p=0.63
Nr0b1 p=0.16
Tet1 p=0.49
Sox2 p=0.07
Nanog p=0.37
Tcl1 p=0.44
B
2i+Serum+LIF
Nanog-Low to Nanog-SH
Total reporter (a.u.)
Time (hours)
* State-switching event
C
By simulation
Up-step frequencies
Down-step frequencies
Burst size
Burst frequency
Total Protein
Time
Burst size (mRNA/burst)
Burst frequency (hour^{-1})
Protein production rate mixing time
Faster mixing
Burst size (# mRNA)
Burst freq. (hour^{-1})
$\tau_{A=0.5}$ norm. (fold change)
Production rate rank
Production rate rank at t = 0
Highest
Lowest
D
Serum+LIF: Nanog-Hi intra-state
2i+Serum+LIF: Nanog-SH intra-state
Total Protein (a.u.)
Production rate (a.u.)
Production rate rank
Time (hours)
Production rate rank at t = 0

Table S1. State-switching events show no correlation between sister cells

Direction of switch \ # of sister pairs*	Neither sister switched	Only one sister switched	Both sisters switched	Expected number of sister pairs that both switched [95% C.I.]**
N^{Lo} to N^{Hi}	169	7	0	**[0 – 1]**
N^{Hi} to N^{Lo}	139	15	2	**[0 – 2]**

Data shown are combined results from two independent experiments. Analysis of individual data sets yields the same conclusion.

* Data points are discarded if one of the cells in a sister pair was lost or not traceable in the movie

** Confidence interval obtained by random permutation test with 100,000 trials. Green indicates observed frequency of sister pairs in which both cells switched falls within the 95% C.I.

Supplemental Figure, Table and Movie Legends

Figure S1. Validation of smFISH; related to Figure 1

(A) A stack of snapshots taken through the whole volume of a single cell; the resulting maximum-intensity projection (green box), and a single slice (blue box) are fed into the image-processing algorithm for dot-detection.

(B) Cumulative Distributions of dot counts for each of the two imaging approaches is shown across a population of cells.

(C) Same distributions as in B, but normalized by the sample median.

(D) Technical replicates for the single-slice approach.

(E) Correlation between dGFP protein fluorescence as measured simultaneously with *dGFP* transcripts. (left), and correlation between *Rex1* (unmodified allele) and *dGFP* (knock-in reporter on second allele) transcripts (right). r is the Pearson correlation coefficient.

(F) (Left) Sorted subpopulations of the bimodal Rex1-*d*GFP knock-in reporter. (Right) qPCR results on these subpopulations for a subset of target genes also examined by smFISH. Values were normalized to expression levels of the housekeeping gene *Gapdh*, and are represented as 2^(-ΔΔCt) with respect to the '*Rex1*-high' subpopulation

Figure S2. mRNA distributions and correlations by smFISH; related to Figure 2

(A) Empirical distributions and MLE fits for unimodal, bimodal, and long-tailed genes. *p*-values are for χ^2 GOF tests. $p>0.05$ indicates that the fit to the distribution is indistinguishable from the empirically measured distribution. Where present, solid lines represent components of the fit. Dashed line represents the overall fit to the distribution.

(B) Pairwise relationships between heterogeneously expressed genes. *p*-values are from the 2D KS-test. *r* is the Pearson correlation coefficient.

(C) Correlation and marginal distributions of *Rex1* and *Nanog* in a control population (top) and population synchronized by a double thymidine block fixed immediately following the block (bottom). r

is the Pearson correlation coefficient.

Figure S3. Differential methylation between *Rex1* states; related to Figure 3

(A) Locus specific bisulfite sequencing plots between *Rex1*-high, -low, and -low-to-high-reverting cells at three targets of methylation. Open circles are unmethylated, filled circles are methylated, and x's are unknown.

(B) Measurements from A are plotted as bar graphs for comparison.

(C) Scatter plots showing how single CpGs in the promoters of a given gene change between *Rex1*-high and -low states. Color coding represents the position of a base relative the transcriptional state site.

Figure S4. Construction and analysis of live cell reporters, and simulations based on observed kinetics; related to Figure 4

(A) Schematic of *Nanog* reporter (top) and *Oct4* reporter (bottom) construction.

(B) Correlation between *Nanog* (unmodified allele) and *Citrine* (knock-in reporter on second allele) transcripts in N_{KI}Cit cell line. r, Pearson correlation coefficient. Light blue, presumed fraction of cells with silenced reporter cassettes (~10% of all cells; see Supp. Info. for discussion); dark blue, remaining cell population.

(C) H2B-Citrine protein degradation rate assayed by blocking translation during movie at time indicated. Total YFP became flat (top) with negligible slope (bottom) shortly after cycloheximide treatment.

(D) Identification of sharp inflections in total fluorescence traces. (i) First, frames around cell divisions are removed and fluorescence lost during divisions is added back to the daughter trace to create a continuous trace for each lineage (ii), where a step detector spanning a 6-hour window is applied across consecutive frames. (iii) For each window, a one-piece linear fit is compared with a two-piece fit that is flexible at the midpoint. A two-piece fit is considered better than a one-piece fit when two criteria are

met: 1) Residual noise of the one-piece fit is higher than a threshold, see Supp. Info.), and 2) the slopes of the two-piece fit are significantly different between the two pieces. iv) For each stretch of frames meeting both criteria 1 (magenta line indicates threshold) and 2 (orange line indicates where two-piece fit yields significantly different slopes), the window with the highest residual noise is assigned to be the inflection. v) Continuized trace approximated into linear segments between identified points of inflection.

(E) Apparent steps from simulated Oct4 expression under the bursty transcription model using parameters estimated from smFISH.

(F) Protein traces were simulated under the bursty transcription model over various mRNA half-life and burst frequency combinations; mean burst size was kept constant at 35 mRNA/burst. Gaussian noise proportional to the total protein level and equivalent to the magnitude of frame-to-frame variation empirically observed was added to the simulated traces for comparability. Arrowheads indicate detected steps on simulated trace of the corresponding color. Note that changes in production rate around cell division events can be identified as steps either before or after the division. Red box: Estimated regime for Nanog-Hi in serum+LIF. Right: Variation in the frequency of detected steps over the same parameter space.

(G) Production rates decrease by an average of 0.63-fold across cell divisions. Each point represents a division event. Average production rates of the 4-hour windows before and after each cell division are compared. Black dotted line: zero change; grey dotted line: 0.5-fold change; purple line: average trend; Inset: example trace indicating slope before and after division.

(H) Changes in production rate over state-switching events or intra-state steps. “Higher rate”-to-“lower rate” ratios are plotted for all steps and events, i.e. down-steps and *Nanog*-high-to-*Nanog*-low switching events are represented by the reciprocals of rate change. (*p*-value, KS test)

Figure S5. Quantitative analysis of how 2i+serum+LIF affect static distributions and dynamics of

gene expression for pluripotency regulators; related to Figure 5

(A) smFISH transcript count distribution of factors in 2i+serum+LIF with MLE fits overlaid. p-values are for χ^2 GOF tests. p>0.05 indicates that the fit to the distribution is indistinguishable from the empirically measured distribution. Where present, solid lines represent components of the fit. Dashed line represents the overall fit to the distribution.

(B) Example trace of cells switching from *Nanog*-low to *Nanog*-SH in 2i+serum+LIF.

(C) Left: simulated traces similar to Fig. S4F, except over various combinations of burst size and burst frequency; mRNA half-life was kept constant at 4 hours. Bottom right: rank of production rate of 30 randomly selected traces (out of a total of 200) in each simulation under the corresponding parameter combination. Traces are color-coded by the initial rank at t = 0 as in D. Top right: mixing time of protein production rate, defined as the time where auto-correlation of rank drops below 0.5.

(D) *Nanog* expression dynamics of cells in serum/LIF with or without 2i. Each trace represents one cell randomly picked from a tracked lineage tree. Production rates are normalized by cell size and ranked within the group for each time point. Traces are color-coded by the initial rank at t = 0.

Table S1. State-switching events show no correlation between sister cells; related to Figure 4

Movie S1. *Nanog*-high to *Nanog*-low switch in serum+LIF; related to Figure 4

Cells imaged in serum+LIF condition. Shown are examples of cells switching from *Nanog*-high to *Nanog*-low.

Movie S2. *Nanog*-low to *Nanog*-high switch in serum+LIF; related to Figure 4

Cells imaged in serum+LIF condition. One of the lineages switched from *Nanog*-low to *Nanog*-high.

Movie S3. *Nanog*-SH cells in 2i+serum+LIF; related to Figure 5

Cells imaged in 2i+serum+LIF condition. Nanog reporter expression is homogeneous compared to cells grown without 2i.

Movie S4. *Nanog*-low to *Nanog*-SH switch in 2i+serum+LIF; related to Figure 5

Cells imaged in 2i+serum+LIF condition. Shown are *Nanog*-low cells which were rare and switched to *Nanog*-SH.

Supplemental Experimental Procedures

Detailed Culture Conditions

All cells were maintained in humidity-controlled chamber at 37ºC, with 5% CO_2 in serum+LIF media [Glasgow Minimum Essential Medium (GMEM) supplemented with 10% FBS (HyClone, Thermo Scientific), 2 mM glutamine, 100 units/ml penicillin, 100 ug/ml streptomycin, 1 mM sodium pyruvate, 1000 units/ml Leukemia Inhibitory Factor (LIF, Millipore), 1X Minimum Essential Medium Non-Essential Amino Acids (MEM NEAA, Invitrogen) and 50 uM β-Mercaptoethanol.

Correlation between *Citrine* and *Nanog* transcripts in *Nanog* knock-in reporter cells (N_{KI}Cit)

We validated the *Nanog* knock-in reporter by performing smFISH for correlation between *Nanog* (unmodified allele) and *Citrine* (knock-in reporter on second allele) (Fig. S4B). We observed that when grown in serum+LIF conditions, ~10% of cells contained *Nanog* but no *Citrine* transcripts, likely due to silenced expression of their reporter cassettes during prolonged propagation without antibiotics. The remaining cell population showed even stronger correlation between *Nanog* and *Citrine* transcripts ($r = 0.78$). We corrected for the potential systematic error that may result in the calculation of low-to-high switching rate such that an observed rate of 1.9 ± 0.29 transitions per 100 cell cycles was adjusted to the reported 2.3 ± 0.25 (mean ± SD). We note that the magnitude of this error does not alter key conclusions, including those about the relative stabilities of the two states. Furthermore, the asymmetry of this silencing behavior – we did not find a corresponding fraction of cells expressing *Citrine* but no *Nanog* transcripts – suggests that this is unlikely a result of mono-allelic regulation.

smFISH Procedure and imaging system

Up to 48 20mer DNA probes per target mRNA were synthesized and conjugated to Alexa fluorophore 488, 555, 594, or 647 (Life Technologies) and then purified by HPLC. Cells for smFISH experiments were plated at 40,000/cm^2 and harvested after 48 hours. Trypsinized cells were washed in PBS and fixed in 4% formaldehyde at room temperature for 5 mins. Fixed cells were resuspended in 70% ethanol and

stored at -20°C overnight. The next day, cells were hybridized with 4nM probe per target species at 30°C, in 20% Formamide, 2X SSC, 0.1g/ml Dextran Sulfate, 1mg/ml E.coli tRNA, 2mM Vanadyl ribonucleoside complex, and 0.1% Tween 20 in nuclease free water. The following morning, cells were washed in 20% Formamide, 2x SSC, and 0.1% Tween 20 at 30°C, followed by two washes in 2x SSC + 0.1% Tween 20 at room temperature. Hybridized cells were placed between #1 coverslips and flattened by applying pressure evenly across the glass.

After flattening cells between coverslips, dots typically span two distinct focal planes. However, to maximize the number of cells imaged in a given acquisition time, only one of these focal planes was captured. This results in approximately ~60% of each cell's transcripts being captured in a single slice, as compared to taking a stack of images across the entire volume of each cell (Fig. S1A-D).

Imaging was performed on a Nikon Ti-E with Perfect Focus, Semrock FISH filtersets, Lumencor Sola illumination, 60x 1.4NA oil objective, and a Coolsnap HQ2 camera. Snapshots were taken using an automated grid-based acquisition system on a motorized ASI MS-2000 stage.

Monte-Carlo Bivariate Kolmogorov-Smirnov Test

The 1D Kolmogorov-Smirnov test was extended to 2D dimensions (Peacock, 1983) to determine whether an empirical bivariate distribution showed any dependence between variables; the 2D Cumulative Distribution Function (CDF) is computed in each possible quadrant of the 2D plane $P(x<x0)$, $P(y<y0)$; $P(x>x0)$, $P(y<y0)$; $P(x<x0)$, $P(y>y0)$; and $P(x>x0)$, $P(y>y0)$. The 2D KS test statistic is thus defined as the largest difference between empirical and theoretical distributions across each of these possible regions. In order to generate a test-statistic distribution under the null hypothesis, we performed a Monte-Carlo simulation where sets of random pairs of data points are sampled from the PDF formed by the product of the marginal distributions. The resulting bivariate CDF is compared to the theoretical CDF and the maximal difference is taken. This is performed repeatedly in order to generate a distribution of

maximal differences that would occur by chance. Finally, the test statistic is computed from the empirical distribution, and compared to this distribution at a 95% confidence level.

Movie acquisition system

Images were acquired on the IX81 inverted microscope system (Olympus) using the Metamorph acquisition software (Molecular Devices) with the iKon Charge Coupled Device (CCD) camera (Andor). Fluorophores were excited using X-Cite XLED1 light source (Lumen Dynamics) equipped with the BLX, BGX and GYX modules.

Movie data analysis: Segmentation and tracking

The Schnitzcells script package (Young et al., 2011) was used to segment and track cells from the acquired images. This package performs a number of procedures as described below. Briefly, cells were segmented with Matlab built-in edge detection script, using Laplacian of Gaussian method. Segmented cells in individual frames were then tracked across all time points by performing a point-matching algorithm on successive pairs of frames to generate a cell lineage data structure. To obtain the total fluorescence level of each cell, the images were "flattened" by correcting for the nonuniformity of illumination, followed by local background correction that takes into account the camera acquisition background, autofluorescence from the medium and fluorescence contribution from neighboring cells.

Movie data analysis: Production rate estimation and step detection

To enable the continuous estimation of production rates (slopes), frames around cell divisions are removed and fluorescence lost during divisions (to sister cells) is added back to the trace of interest to create a continuous total fluorescence trace for each lineage. Instantaneous fluorescence production rates were estimated by fitting the continuous total fluorescence of a 6-hour window to a linear section using the linear least squares method. Distributions of reporter production rates (Figs. 4A,B) were obtained by sampling the instantaneous fluorescence production rates of all cell lineages at 1-hour intervals. To

characterize abrupt changes in production rates, we identified sharp inflections of the continuous total fluorescence traces by applying a custom-built step detector on overlapping and consecutive 6-hour windows 15 minutes apart (Fig. S4D). For each window, we obtained fits to a linear polynomial model and a continuous two-piece linear polynomial model with a joint at midpoint using the linear least squares and non-linear least squares methods, respectively. The continuous two-piece linear model can be represented as follows:

$$y = \begin{cases} m_a x + c & , \quad x < x_{mid}, \\ m_a x_{mid} + c + m_b(x - x_{mid}) , & \quad x \geq x_{mid}, \end{cases}$$

where x_{mid} is the midpoint of the window.

We used two criteria to determine whether a given window fits better to the one-piece or two-piece linear fits: (1) whether the noisiness of the trace can explain the deviation of the data from the one-piece fit (mean sum of squared errors, M.S.E.), and (2) whether the two slopes obtained from the two-piece fit are significantly different from each other. For (1), we define the noisiness of the trace as the variance of the distribution of frame-to-frame fluctuations in total fluorescence, i.e. $var(Y_{t+1} - Y_t)$. For a perfectly linear trace without noise, the mean of $Y_{t+1} - Y_t$ equals the slope of the trace. As the observation noise increase, the SSE of one-piece fit increases even if the underlying trace has a constant slope. We therefore estimated the portion of SSE of one-piece fit unexplained by the noisiness of the trace as the residual noise, defined as $MSE_{1pc} / var(Y_{t+1} - Y_t)$, where *n* is the number of frames within a window. For (2) we obtained the 95% confidence bounds of the two slopes in the two-piece fit and determined if they overlap. Using (1) and (2), we identified stretches of frames where two-piece fit is significantly better than one-piece. The frame with the highest residual noise among each of these stretches was designated as the point of inflection and the rest of the trace was approximated by linear segments between these points.

Movie data analysis: Hidden Markov Model and Viterbi Algorithm

We set up a two-state HMM to estimate the frequency of state-switching events between the higher and

lower Nanog states. We assume each of the two states can produce an independent Gaussian distribution of production rates, with specified mean and variance, including potential overlap between the two states. Over each unit time, a cell can either stay at its current state or switch to the other state with specified probabilities. Thus, given a specific parameter set, there exists for the production rate time-series of each cell a corresponding series of underlying states that has the maximum likelihood. This likelihood is a balance between the probability of observing a production rate at the corresponding state and that of switching to another state, such that a cell that transiently exhibits a production rate far from the mean of its current state is more likely to be fluctuating rapidly within a state than switching away and back. The Baum-Welch algorithm (Do et al., 2008) maximizes the sum of this likelihood over all cells by iteratively changing the parameters in small increments, improving the total likelihood each time.

Prior to training the model with data, initial transition rates between the states in both directions were set at 0.0001/hour. Initial parameters for each state were set with the mean value drawn from the range of observed production rates and variance. Re-initializing the random parameters in the model yielded similar results. We employed the HMM toolbox for Matlab (Murphy, 1998), which generated maximum likelihood estimate of the model parameters using the Baum-Welch algorithm. Since the production rate sequences used to train the HMM contained repeated time-series when multiple lineages shared the same ancestor, the state-transition rates generated directly from HMM could be an overestimation. We applied the Viterbi algorithm (Rabiner, 1989) to combine the model parameter estimates and the observed data to infer the most likely state sequence for each cell lineage. From this we reported the empirical state-transition rates, normalized to the average length of a cell cycle.

Bursty transcription simulation and mixing time analysis

Bursty transcription was simulated using the model previously described (Peccoud and Ycart, 1995). In this model, a promoter can transit stochastically between an active and an inactive form. This is not to be confused with a cellular state, which is usually maintained over a longer timescale and within which a

gene bursts in a characteristic burst size and frequency. Transcription occurs only when the promoter is in its active form, producing a burst of mRNA molecules, which decay exponentially. To aid comparison between the simulated transcription dynamics and our experimental observations, we added protein production to the simulation. Further, since our fluorescence protein is stable, and to restrict the source of heterogeneity in our simulation to stochastic transcription, we assumed zero protein decay rate and deterministic protein production at a constant rate. Lastly, both mRNA and protein are partitioned when cells divide, which were set to have division rates similar to experimental data. This model can be described by the following reactions:

$$
\begin{aligned}
I &\xrightarrow{k_{on}} A \\
A &\xrightarrow{k_{off}} I \\
A &\xrightarrow{\beta_m} A + m \\
m &\xrightarrow{\alpha_m} \emptyset \\
m &\xrightarrow{\beta_p} m + p
\end{aligned}
$$

Here, A and I denote the promoter in its active and inactive forms, respectively; m -- mRNA level; p -- protein level; k_{on} and k_{off} -- activating and inactivating rates of the promoter, respectively; α_m -- mRNA degradation rate; β_m -- mRNA production rate; Ø -- mRNA degradation; β_p -- protein production rate.

A cellular state is thus characterized by the frequency of mRNA bursts and the mean number of mRNA molecules produced per bursts. Here, we considered one limiting case of this model, where k_{off} is significantly larger than k_{on} and somewhat larger than α_m. This assumption can be related physically to a scenario where bursts are relatively infrequent and have short durations, and the distribution of mRNA levels produced under these assumptions can be described with a single gamma (Raj et al., 2006) or NB function (Paulsson and Ehrenberg, 2000). A cell changes state in a gene when one or more of the parameters k_{on}, k_{off} or β_m for that gene is changed, thus resulting in different burst frequencies and sizes.

To simulate mRNA and protein dynamics for the *Nanog*-high state in serum+LIF condition (shown in

Figs. 4E, S4E), we used the following parameters estimated from mRNA distributions in smFISH: For *Nanog* -- burst size = 33 mRNA/hour, burst frequency = 0.39 bursts/hour; For *Oct4* -- burst size = 87 mRNA/hour, burst frequency = 0.52 bursts/hour. These assume that mRNA half-lives of *Nanog* and *Oct4* are 5.85 and 7.4 hours, respectively (Sharova et al., 2009).

We utilized computer simulations of this model to explore whether changes in state affect the intra-state dynamics of heterogeneity. Varying burst frequency and burst size results in traces with various frequencies of apparent steps when analyzed using the same step detector, which identified regimes in the bursty transcription parameter space where steps of similar quality to the ones observed can be generated (Figs. S4F, S5C). Furthermore, the resulting dynamics also display a wide range of shapes of fluctuation and levels of expression. We quantified these variations with an objective measurement, the "mixing time", a population metric adapted from Sigal et al. (2006). For each simulated population (n = 200 traces) using a single parameter set, we ranked all traces by their production rate at each time point. Thus a cell starting with the lowest production rate among the population may change in this rank when its production rate changes over time. We computed the autocorrelation function $A(\tau)$ of this rank for each population and the mixing time is defined as the time lag τ at which $A(\tau)$ decayed to 0.5. We opted to calculate the mixing time using production rate but not total fluorescence level because the stable fluorescent reporter facilitates accurate production rate estimate but may not reflect the physiological level of endogenous proteins. Additionally, for more direct comparison between the mixing times calculated from simulated and observed data, the production rates in simulation were computed using the simulated protein traces after Gaussian noise similar to the level observed was added.

Supplemental References

Do, C.B., and Batzoglou, S. (2008). What is the expectation maximization algorithm? Nature Biotechnology 26, 897–899.

Murphy, K.P. (1998). Hidden Markov Model (HMM) Toolbox for Matlab. Retrieved Dec 6, 2012, from http://www.cs.ubc.ca/~murphyk/Software/HMM/hmm.html.

Rabiner, L.R. (1989). A tutorial on hidden Markov models and selected applications in speech recognition. Proceedings of the IEEE *77*, 257–286.

Sharova, L., Sharov, A., Nedorezov, T., Piao, Y., Shaik, N., and Ko, M. (2009). Database for mRNA half-life of 19 977 genes obtained by DNA microarray analysis of pluripotent and differentiating mouse embryonic stem cells. DNA Research *16*, 45.

Young, J.W., Locke, J.C.W., Altinok, A., Rosenfeld, N., Bacarian, T., Swain, P.S., Mjolsness, E., and Elowitz, M.B. (2011). Measuring single-cell gene expression dynamics in bacteria using fluorescence time-lapse microscopy. Nature Protocols *7*, 80–88.
